# Influence of urban forests on residential property values: A systematic review of remote sensing-based studies

**DOI:** 10.1016/j.heliyon.2023.e20408

**Published:** 2023-09-24

**Authors:** Ewane Basil Ewane, Shaurya Bajaj, Luisa Velasquez-Camacho, Shruthi Srinivasan, Juyeon Maeng, Anushka Singla, Andrea Luber, Sergio de-Miguel, Gabriella Richardson, Eben North Broadbent, Adrian Cardil, Wan Shafrina Wan Mohd Jaafar, Meshal Abdullah, Ana Paula Dalla Corte, Carlos Alberto Silva, Willie Doaemo, Midhun Mohan

**Affiliations:** a United Nations Volunteering Program, via Morobe Development Foundation, Lae 00411, Papua New Guinea; b Ecoresolve Inc., San Francisco, CA, USA, 94105; c Department of Geography, Faculty of Social and Management Sciences, University of Buea, P.O. BOX 63 Buea, Cameroon; d Unit of Applied Artificial Intelligence, Eurecat, Centre Tecnològic de Catalunya, 08005 Barcelona, Spain; e Department of Agricultural and Forest Sciences and Engineering, University of Lleida, Av. Alcalde Rovira Roure 191, 5198 Lleida, Spain; f Department of Forest Analytics, Texas A&M Forest Service, Dallas, TX 75252, USA; g AAP Labs, Cornell University, USA; h Joint Research Unit CTFC-AGROTECNIO-CERCA, Ctra. Sant Llorenç de Morunys km 2, 25280 Solsona, Spain; i Department of Sociology and Anthropology, University of Guelph, Guelph ON, Canada; j Spatial Ecology and Conservation (SPEC) Lab, School of Forest, Fisheries, and Geomatics Sciences, University of Florida, PO Box 110410, Gainesville, FL 32611, USA; k Tecnosylva, S.L Parque Tecnológico de León, 24004 León, Spain; l Earth Observation Centre, Institute of Climate Change, Universiti Kebangsaan Malaysia, Bangi 43600, Selangor, Malaysia; m Department of Geography, College of Arts and Social Sciences, Sultan Qaboos University, Muscat, P.O. Box 50, Oman; n Department of Ecology and Conservation Biology, Texas A&M University, College Station, TX, USA; o BIOFIX Research Center, Federal University of Paraná (UFPR), Curitiba 80210-170, Brazil; p Forest Biometrics, Remote Sensing and Artificial Intelligence Laboratory (Silva Lab), University of Florida, USA; q Department of Civil Engineering, Papua New Guinea University of Technology, Lae, 00411, Papua New Guinea; r Morobe Development Foundation, Doyle Street, Trish Avenue-Eriku, Lae 00411, Papua New Guinea; s Department of Geography, University of California-Berkeley, Berkeley, CA 94709, USA

**Keywords:** Urban tree planting, Urban forests and climate change, Remote sensing, GIS, Urban forests benefits, Housing prices, Urban trees

## Abstract

Urban forests provide direct and indirect benefits to human well-being that are increasingly captured in residential property values. Remote Sensing (RS) can be used to measure a wide range of forest and vegetation parameters that allows for a more detailed and better understanding of their specific influences on housing prices. Herein, through a systematic literature review approach, we reviewed 89 papers (from 2010 to 2022) from 21 different countries that used RS data to quantify vegetation indices, forest and tree parameters of urban forests and estimated their influence on residential property values. The main aim of this study was to understand and provide insights into how urban forests influence residential property values based on RS studies. Although more studies were conducted in developed (n = 55, 61.7%) than developing countries (n = 34, 38.3%), the results indicated for the most part that increasing tree canopy cover on property and neighborhood level, forest size, type, greenness, and proximity to urban forests increased housing prices. RS studies benefited from spatially explicit repetitive data that offer superior efficiency to quantify vegetation, forest, and tree parameters of urban forests over large areas and longer periods compared to studies that used field inventory data. Through this work, we identify and underscore that urban forest benefits outweigh management costs and have a mostly positive influence on housing prices. Thus, we encourage further discussions about prioritizing reforestation and conservation of urban forests during the urban planning of cities and suburbs, which could support 10.13039/100004420UN Sustainable Development Goals (SDGs) and urban policy reforms.

## Highlights

• There exists a paucity of Remote Sensing (RS) studies focusing on developing countries
• 39.3% of the RS studies were conducted in the U.S., 32.6% in China, 4.5% Australia and 13.5% in Europe
• Tree cover, forest size, type and greenness were the primarily used metrics
• Increased urban forest presence mostly had a positive effect on housing prices
• Satellite and aerial imagery were the most used RS data sources

## Graphical abstract

**Figure:**
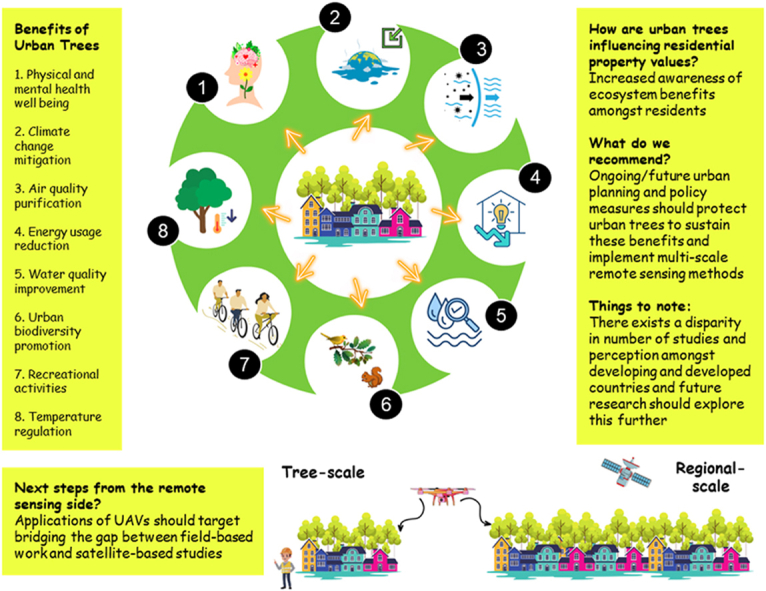

## 1 Introduction

### 1.1 Research background

Urban forests can be commonly classified as (i) peri-urban forests and woodlands surrounding towns and cities, (ii) city parks and urban forests >0.5 ha, (iii) pocket parks and gardens with trees <0.5 ha, (iv) trees on streets and in public squares, and (v) other green spaces with trees such as riverbanks, vacant lands [1]. Residential properties comprise standalone single-family housing, townhouses, and multi-family housing apartments, including low-rise garden apartments and mid- and high-rise apartments, and any other buildings where people live or dwell in cities and suburbs. The existing literature provides concrete evidence of relationships between urban forests and residential property values. Urban forests provide multiple benefits to human well-being and the environment and contribute directly and indirectly to the achievement of nine UN sustainable development goals (SDGs) [1]. Thus, understanding the functional relationship between urban forests and residential property values is invaluable for sustainable urban planning and management to improve human well-being [[2], [3], [4], [5]].

Urban forests provide benefits to the population through regulating ecosystem services (ES) such as pollution control, water quality regulation, carbon sequestration, noise reduction, and microclimate regulation, coupled with recreation and aesthetics cultural services [3,[5], [6], [7], [8], [9], [10]]. These ecosystem services have been capitalized by housing markets into prices that drive the effect of urban forests on residential property values and can be further tapped through appropriate urban planning measures that promote urban forests [9,11].

In addition to the presence of urban forests, residential property values also depend on several factors, including the type of environmental attributes, neighborhood attributes, urban forest conditions, and property structural attributes. Nonetheless, the outcomes are highly variable [9,12,13]. Forest attributes such as larger mature trees with medium canopy closure [6,13] and extensive forest cover closer to residential properties have a positive effect on housing prices, especially where increased public surveillance is implemented to lower associated crime rates [3,6,9,10,[14], [15], [16]]. Thus, upscaling tree planting in urban areas to acceptable thresholds has a positive impact on climate change.

For developing countries, urban forests' effect on residential property values is mostly reflected for homeowners but not necessarily for home renters, because buying a house is a long term capital investment decision compared to renting a house. Home renters prioritize to satisfy property structural attributes first, like house size, than to invest more due to distance to and size of urban green spaces [9]. However, the local population is increasingly assigning willingness to pay for the associated costs of ecological restoration of non-developed natural areas [9,17,18] and the development of new neighborhood parks since people are becoming more aware of the direct benefits of urban green spaces [19].

However, non-developed urban forest areas buffering unattractive land uses such as industrial areas even exacerbate the negative influence on housing prices nearby [12,20,21]. Non-developed urban forests offer fewer recreational and aesthetic opportunities to positively sway housing prices [3], particularly in developing countries [9]. Urban forests in areas prone to natural disturbances such as wildfires, pest outbreaks, and windthrow can negatively affect the property value and broader housing market as it lowers the physical condition of the urban forest amenities and property itself [21,22]. Excessive tree cover and insufficient investments in the management of tree cover in a property or a residential neighborhood may become a disamenity and nuisance to the people and community [11]. Urban tree planting to improve access to urban green spaces by the population may lead to gentrification and displacement of current lower-income residents because of the increase in housing prices in the area [23].

Therefore, knowledge of urban forest and tree cover characteristics requirements in the residential property context, and the protection of neighborhood ecological and social sustainability is key for timely urban planning practices and urban policy reforms. Such knowledge is invaluable to inform urban reforestation programs and local regulations promoting urban forests and trees [6,24], and avoiding gentrification and displacement of low-income people due to increased house prices [9].

Most studies used a combination of the hedonic valuation model and econometric analysis to capture and quantify the proportion of the economic value of urban forest characteristics on residential property values by controlling for the effect of neighborhood/locational and property structural attributes. Urban forests characteristics such as tree cover, tree density, tree biomass, leaf area index (LAI), vegetation greenness [6,10,11,13,16,[24], [25], [26], [27], [28]] and, proximity to and size, type, and condition of forests [3,9,10,24,[29], [30], [31]] were used in hedonic models. Other studies have used vegetation greenness based on the normalized difference vegetation index (NDVI) in hedonic models in relation to housing prices [10,28,[32], [33], [34], [35], [36]].

Few studies have also used the contingent valuation model to account for urban forests' effect on residential property values [[37], [38], [39]]. This is because many of the regulating and supporting benefits of forests are less suitable for hedonic valuation models due to a lack of accurate measures of individual ES variables and understanding and recognition of ES by housing buyers [3], such that novel valuation and measurement methods need to be integrated [40].

Urban forests structure and tree canopy mapping and assessments are invaluable for understanding specific urban forests characteristics influencing residential property prices. Field measurement of urban forests and tree characteristics in random sample plots designs have been used to estimate the effect of urban forest structure on residential property values [6,26]. However, field surveys are cost-, time- and labor-intensive, especially over larger spatial scales, and only allows for the visual estimation of greenness and tree-level metrics related to forest cover. Remote sensing approaches are good complementing strategies to field surveys to improve our understanding of the influence of urban forests on residential property values. Thus, high resolution remote sensing data and GIS (Geographic Information System) have been used for urban forests and tree-level mapping and assessment to quantify the economic value of urban forests on residential property values in hedonic models, especially when integrated with well-established field surveys [9,10,12,13,21,28,30,41,42].

### 1.2 Research gap

Remote sensing offers an opportunity for better spatial and temporal analyses of forest metrics, vegetation indices, and forest regeneration over larger areas [6,10,43]. The accuracy of remote sensing-derived forest, tree and vegetation metrics have been improving with the advancements in machine learning algorithms combined with multi-temporal LiDAR (Light Detection and Ranging) point clouds and UAV (Unmanned Aerial Vehicles) images. It also allows for extracting finer details, patterns, and changes in information on forests, tree-level and vegetation characteristics and indices with lower costs [[44], [45], [46], [47]]. Remote sensing-derived forests and tree-level data are important input variables for inclusion in hedonic pricing models to improve our understanding of the relationship between urban forests and residential property prices. There are few remote sensing-based urban forests and ecosystem services mapping and assessment studies that provide suitable variables for input in hedonic pricing models to improve our understanding of the relationship between urban forests and residential property values [3,9].

### 1.3 Aim and contribution of the study

The aim of this review is to provide important insights on how using remote sensing tools and data sources, respectively, to map and assess urban forests and tree-level characteristics can better improve our understanding of the influence of urban forest/trees on residential property values. In particular, we synthesized our findings to draw general inferences on: i) the geographical focus and distribution of the studies, ii) the direction of urban forests/trees and residential property values relations, iii) the remote sensing data sources/types used to measure forest-and tree level parameters, iv) the urban forest/tree-level parameters, vegetation indices and estimation methods/models of urban forests and residential property values relations, and v) the major trade offs in terms of urban forest ecosystem disservices and relations with residential property values. The importance of remotely sensed forest/tree parameters and vegetation indices in improving estimates of the economic value of urban forests effect on residential property values in hedonic models, taking into consideration different urban spatial scale and geographical scale differences is discussed. The results and suggestions shared are expected to help urban policymakers, landscape architects, property developers, and other relevant audiences to implement novel climate-friendly green infrastructure strategies involving restoring and conserving urban forests, benefiting both residential dwellers and housing markets, while explicitly protecting urban ecological, cultural and social sustainability.

## 2 Methods

### 2.1 Data collection

The systematic literature review was performed based on the Preferred Reporting Items for Systematic Reviews and Meta-analysis statement (PRISMA) [48]. The Science Citation Index Expanded/Core Collection Database of Web of Science (WoS), Scopus (https://www.scopus.com/search), and Google Scholar (https://scholar.google.com/) comprised the databases which were searched for relevant literature. Google Scholar search was performed in Python3 using the SerpAPI Google Scholar API (https://serpapi.com/search?engine=google_scholar). Ten Google Scholar pages were consulted for the results of each of the search expression combinations. The results from all the databases were then compiled to form a comprehensive list of peer-reviewed journal articles. The search expressions were created by combining several keywords with the Boolean operators "AND" and “OR”, which identifies the material to be searched in the WoS and Scopus bibliographic databases and Google Scholar search engine. The search period was constrained from the start of January 2010 to the end of September 2022 (Table 1), and was undertaken from July 2022 to September 2022.

**Table 1:** Search expressions utilized in literature review for querying the database.

Criteria	Search Expression
What	“Urban forest” OR “Urban trees”
AND	“Residential” OR “Housing”
AND	“Property value” OR “Housing price”
How	“Remote sensing” OR “GIS” OR “LiDAR” OR “Satellite” OR “Drone” OR “UAV”
When	January 2010 to September 2022

**Fig. 1:**
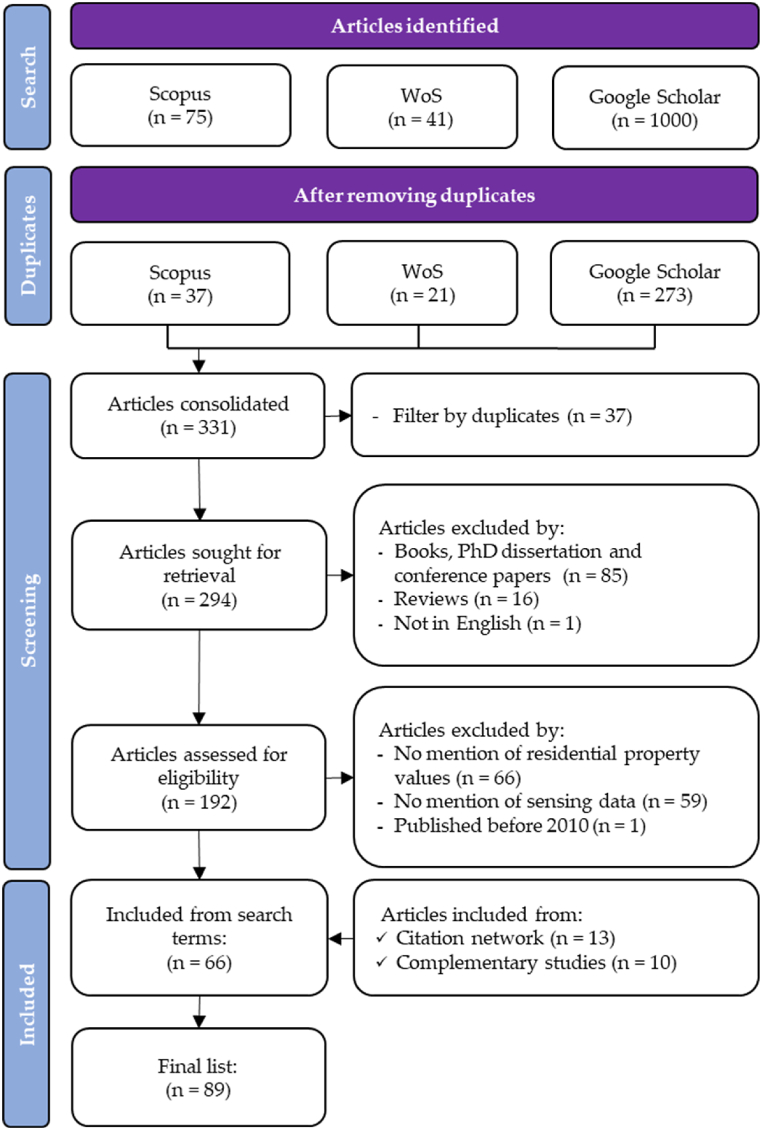
Workflow representing the systematic literature review process.

The relevant aggregated results from the WoS, Scopus, and Google Scholar were 41, 75, and 1000, respectively (Fig. 1). Duplicate results were then removed, which resulted in 294 unique records Further exclusion of books, dissertations, conference, and review papers resulted in 192 collected references, which were then assessed for eligibility according to the following the inclusion or exclusion evaluation criteria, consistent with the Population, Intervention, Comparator, Outcome, and Study design (PICOS) as follows.

1) The study focuses on urban forests, including studies investigating urban green infrastructure, urban green vegetation, and various urban parks and green spaces.
2) The study focuses on residential property value. Studies on rent values, land values, and other property values are excluded.
3) The study is conducted using RS data such as LiDAR, satellite and aerial imagery, UAV or drones, and ground-level images and GIS data.
4) The study clearly states its finding on the influence of urban forests or trees on residential property values.
5) The article is written in English.
6) The article is peer-reviewed.

Finally, a total of 66 peer-reviewed papers were identified based on the defined eligibility criteria (Table 1). In addition, through a citation network analysis, 13 relevant articles that were not obtained as a result of the bibliographic search were included. Later on, 10 new articles, which were identified during the analysis process, were added to the list (Fig. 1). The final list of references contained 89 peer-reviewed papers in total (*See Supplementary Material Reference List*).

### 2.2 Data analysis

We completed a full-text review of the 89 papers, extracted the desired information and provided a descriptive analysis of the systematic review results according to the distribution of papers across countries and cities, geographic distribution of the studies, remote sensing data sources, forest and tree-level characteristics measured, and estimation methods/models. In addition, we extracted information on the influencing ecosystem services, number of publications per year across countries and remote sensing data sources, and the direction (positive or negative) of the relationship between urban forests on residential property values and/or urban human lifestyle at different spatial scales (property level and neighborhood level). The 89 papers of the systematic review were published in 38 academic peer reviewed journals across the globe; mainly in the Landscape and Urban Planning (12), Urban Forestry and Urban Greening (11), Sustainability MDPI (09), Ecological Economics (06), Forest Policy and Economics (05) journals, amongst others (*See* Supplementary Material Table S1 *for details*).

## 3 Results

### 3.1 Geographical focus and distribution of studies

**Fig. 2:**
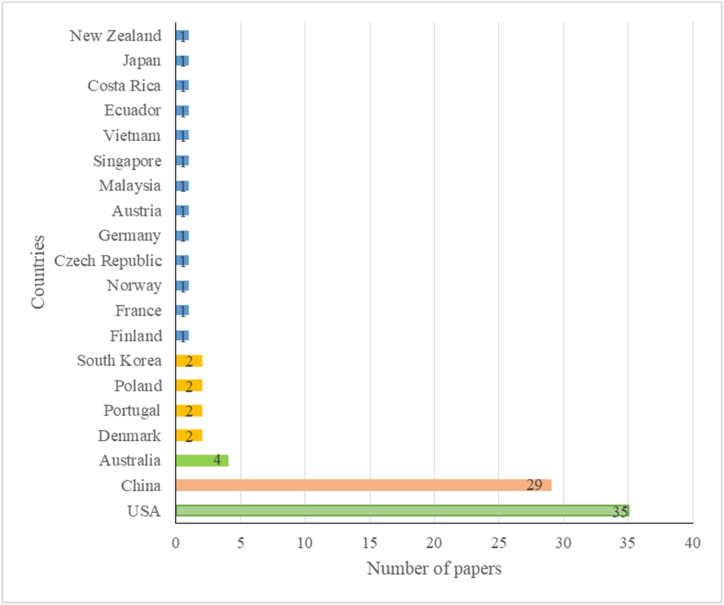
Number of studies for each country as per results of the literature review.

While conducting the literature review, cities were considered to be large metropolitan areas with large populations, vibrant businesses and extensive cultural landscapes, while other urban areas were seen as suburbs, that is, small towns and peri-urban non-rural areas bordering cities within a municipality over a defined boundary based on the degree of urbanization. The review included studies from a total of 21 countries (Fig. 2). Most of the studies were undertaken in cities and suburbs in the U.S. (n = 35; 39.3%), followed by China (n = 29; 32.6%), Australia (n = 4; 4.5%) and Europe (n = 12; 13.5%) for the years 2010–2022, ranking as the first, second and third, respectively.

**Fig. 3:**
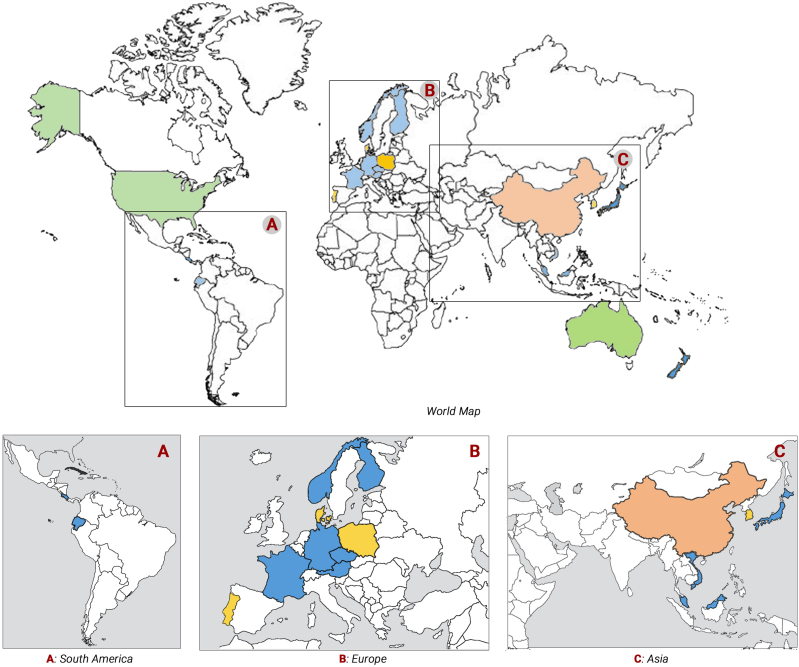
Distribution of studies with respect to the geographical regions.

The distribution of the papers across geographical regions and countries is presented in Fig. 3. About 17 of the 35 studies in the USA were conducted in various cities, while the remaining 18 studies were conducted in suburbs or counties. For Latin America, one study each was completed in Ecuador and Costa Rica (Fig. 3a). For nine European countries, only one study each was conducted in Finland, France, Norway, Czech Republic, Germany, Austria, and two studies each in Denmark, Poland, and Portugal during the considered period (Fig. 3b). For Asia, 29 studies were conducted in 13 cities in China, two studies were undertaken in South Korea and one study each in Malaysia, Japan, Vietnam, and Singapore. For Oceania, four studies were conducted in Australia and one in New Zealand (Fig. 3c). The remaining 54 (of the total of 89) studies conducted in other countries were mostly in cities, except for one conducted in Mosina municipality in Poland. No study was found for developing countries of Africa (Fig. 3).

### 3.2 Types of forest/tree-property relationships and benefits of urban forests

The reviewed studies on urban forests and residential property values using remote sensing technologies produced highly varied and contrasting results at the residential lot and neighborhood scale. The observed variability in the reviewed results was attributed to differences in measured factors such as forest cover, proximity, forest size, accessibility, connectivity, and viewshed of forest and greenery. The majority of the reviewed remote sensing studies considered urban forests and residential property values relationship at the neighborhood scale (*See* Supplementary Material Table S2 *for details*) compared to the residential lot scale (*See* Supplementary Material Table S3 *for details)*. Some of the studies considered urban forests impact on residential property values at both the property and neighborhood spatial scales.

At the residential property scale, tree canopy coverage was reported to have a positive effect on residential property values [16,29,[49], [50], [51]], particularly for middle-to high-income homes with large lots [52]. At the neighborhood public space scale, several studies reported a positive effect of large forest/tree canopy coverage/green vegetation cover within a radius buffer of 20 m–400 m (e.g., Refs. [13,24,49,53]), 500 m–800 m (e.g. Refs. [[54], [55], [56]], and 1 km–3 km [28,[57], [58], [59]] on housing prices (Supplementary Material Table S2).

In addition, other studies focused on grass cover reported a positive effect of proximity to the nearest moderate grassland area within 800 m of the property [19] and parcel and neighborhood irrigated grassland [34,42] on housing prices, while non-irrigated grass cover on the property had a negative effect on house prices [42]. Residents’ high horizontal view of green vegetation within minutes [60], 400 m [61], 160.9 m [62], 253 m [63] of the property were also reported to have positive effects on residential property values. Likewise, proximity to a national park had a positive effect on housing prices [64].

Tree canopy coverage within a residential property was reported to have a negative effect on residential property values in the majority of cases [24,25,42,50,65,66], particularly in low-income neighborhoods [16,52]. A few studies found tree cover within residential properties to have a non-significant effect on housing prices [34,53,67]. Furthermore, at the neighborhood public space scale, few studies reported negative effects of proximity to tree canopy coverage within a 500 m–1000 m radius [13], green vegetation [32], small nearby forest and green spaces [68,69], tree density [70], and distant forest parks of about 5.9 km [58] on housing prices *(*Supplementary Material Table S3*).*

Several studies investigated the effect of vegetation structure, such as the relative importance of trees, trees, and shrubs, shrubs, grass and herbs (irrigated and non-irrigated grass) on residential property values at both parcel and neighborhood levels [19,34,42,51,53,55,59,71,72]. Other studies investigated the influence of vegetation types (percentage of managed vegetation, spontaneous vegetation, and high conservation value forest (HCVF) vegetation) [57], and that of tree type (evergreen, deciduous, native, edible, allergenic, ornamental trees) [70], trees, shrubs and herbs [59] and broad-leaved trees [66] on individual housing prices. One study investigated the influence of urban forest fragmentation (number and size of patches) on housing prices [56]. No study was found to focus on specific trees of major importance in urban landscapes or preferred urban forest tree species suitable for different geographical locations.

**Fig. 4:**
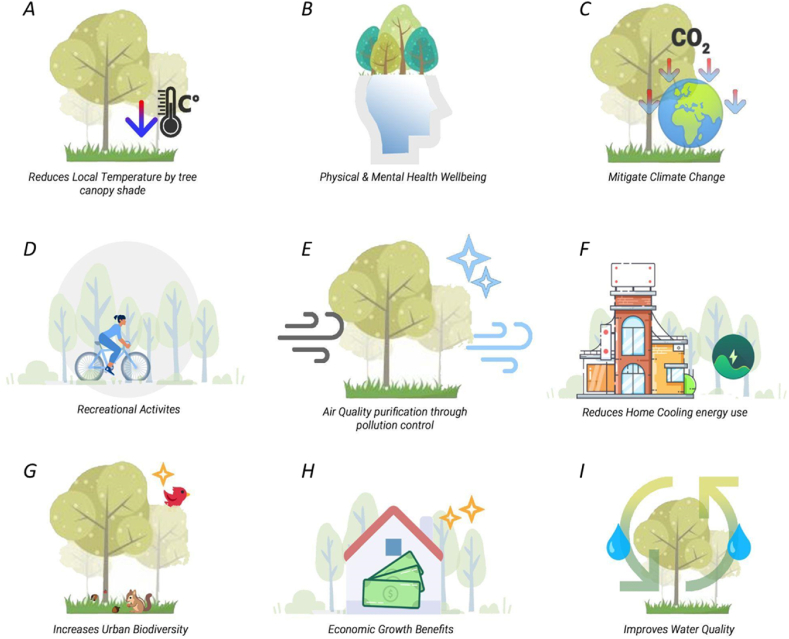
Major ecosystem service benefits of urban forests that positively influence residential property values: a) temperature regulation, b) better health and wellbeing, c) climate change mitigation, d) recreational activities, e) air quality improvement, f) energy usage optimization, g) biodiversity conservation, h) economic growth, and i) water quality improvement.

Reviewed studies directly or explicitly focused on the economic valuation of urban forest benefits in the form of ES and ecotourism on residential property prices were few; most of them were on recreation and aesthetics (96.3%). These were in the form of green viewing, hiking, exercising, biking, and walking, reduced heat due to improved local cooling temperature by shade from tree canopy coverage (74.1%), reduced home cooling energy use (44.4%), and air quality purification (20.4%) in the form of pollution control [3,69,73] within a maximum of 1 km buffer. The key urban forests ES found in the reviewed papers to have maximum impact on residential property prices are illustrated in Fig. 4. The indirect ecotourism benefit of urban forests was covered only in one article by Ref. [10]. However, the results on the perceived impacts of urban forests on residential properties varied highly among residents in different cities/urban areas and countries.

### 3.3 Remotely-sensed data sources

**Fig. 5:**
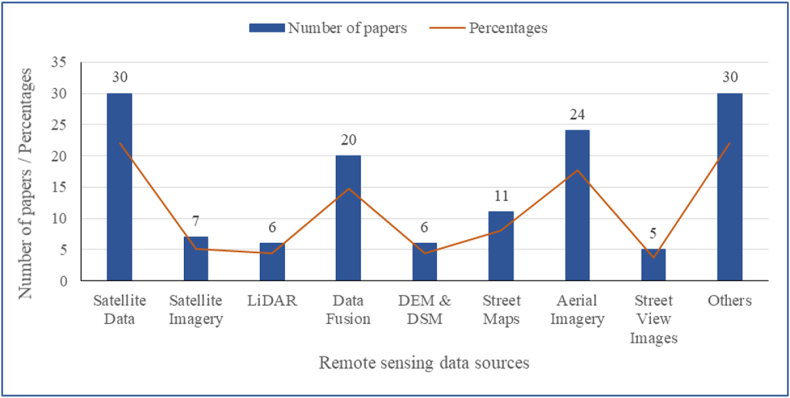
Different sources of remotely-sensed data utilized across various studies.

In the reviewed papers, the authors predominantly used satellite data (n = 30; 21.1%) or aerial imageries (n = 24; 17.6%) or derived byproducts to classify land cover, forest/tree cover, and/or vegetation indices or obtained data (Fig. 5). The satellite data includes Landsat, Quickbird, Advanced Land Observation Satellite (ALOS), and GeoFen-1. Online street maps such as Baidu, Tencent, Google Earth, Open Street Maps, and AMAP (https://ditu.amap.com) were used in 11 (8.1%) of the studies. Five papers (3.7%) reported vegetation and tree cover data based on street view images. Three dimensional data obtained from topographic maps such as Digital Elevation Model (DEM) and Digital Slope Model (DSM) in 6 studies (4.4%) and LiDAR (4.4%) were also used in some 6 case studies.

The use of more than one source of data (data fusion) was observed in 20 (14.7%) of papers. A total of 30 (22.1%) of papers utilized GIS or other data retrieved from government or private sources without specifying remotely sensed data used to generate the data. Most of the studies made use of GIS data, including land use maps, parcel data, census data, boundary data, and more. No study was found that utilized UAV imagery to measure urban forests and vegetation parameters and estimate their influence on residential property values. This might be because of the policies and regulations (e.g., no-fly zone/airspace restriction) restricting the flying of drones over private properties to collect data, such as on urban forests and related vegetation, forest, and tree parameters in many countries and cities.

### 3.4 Urban forest and tree-level parameters, vegetation indices and estimation methods/models

**Fig. 6:**
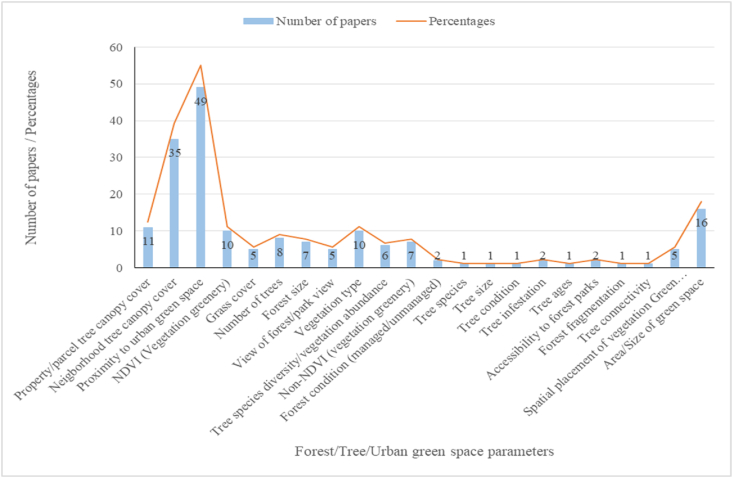
Urban forest/tree/green space parameters measured and the corresponding number of papers.

Remote sensing data sources were used to estimate urban forest (forest size, forest type, greenness/forest condition, proximity, etc.) and tree level (tree canopy cover, tree density, number of trees, tree species, tree condition, tree height, tree size, tree biomass, etc.) parameters. Prominent and widely used forest and tree parameters were identified as neighborhood tree canopy cover (n = 35; 39.3%), area/size of green space (n = 16; 18%), proximity to urban green spaces (n = 49; 55.1%), parcel tree canopy cover (n = 11; 12.4%), and the number of trees within a property (n = 8; 9%), vegetation greenness (n = 10; 11.2%), vegetation type (forest, shrubs, herbs, grass cover) (n = 10; 11.2%), and forest size (large, medium and small) (n = 7; 7.9%) (Fig. 6). The sole vegetation index that was used to measure vegetation greenness (n = 10; 11.2%) was the normalized differential vegetation index (NDVI).

**Fig. 7:**
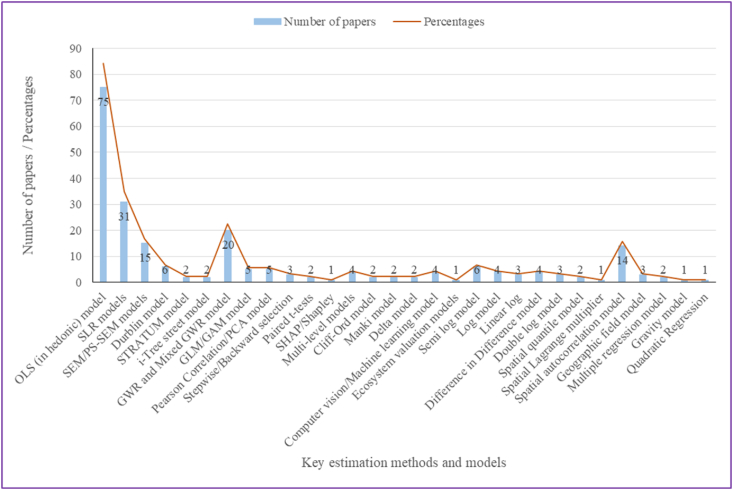
Key methods and models utilized across the various studies to estimate residential property values.

The estimation methods primarily used in the reviewed studies on urban forests and residential property values using remote sensing include the traditional ordinary least square (OLS) model (84.3%), spatial lag regression (SLR) models (34.8%), spatial error model (SEM) including the spatial mixed and fixed effect models (16.9%), the geographically weighted regression (GWR) and mixed GWR models (22.5%), and spatial auto-correlation model (15.7%) mostly in hedonic property pricing models. Only one study used a combination of ecosystem valuation models (e.g., Tree Carbon Calculator, urban-based allometric equations, etc.) to monetize urban forest amenity values [73]. Fig. 7 represents the parameters/indices/methods/models utilized across the reviewed studies.

### 3.5 Major trade-offs

**Fig. 8:**
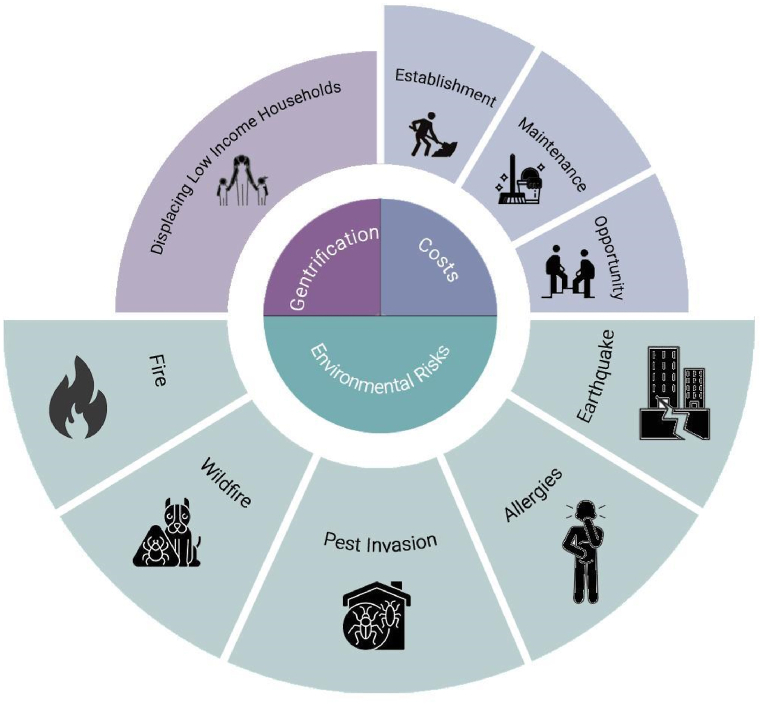
Observed limitations, controversies, and trade-offs associated with urban forests.

Despite the benefits and amenities provided by urban trees and forests, they are also associated with a substantial cost for establishment and maintenance [42,53,66] (Fig. 8). The cost-benefit varies for each situation. For example, in downtown Los Angeles, the tax revenues expected from increased property value from urban green spaces, including trees, shrubs, and grasses, cannot underwrite the cost of neighborhood greening [68], while in Portland, Oregon, the costs are likely to be justified by the increased investment in urban forestry [74]. Even if the benefits outweigh the cost, the positive externalities of trees do not incentivize the property owners to bear the cost of planting and maintaining the trees. The public can enjoy some of the benefits of green spaces established and maintained by private owners, such as air quality improvement or carbon sequestration. The private benefits, such as shade and the resultant energy savings, might be an incentive for single-family detached homes but inconsequential for multifamily properties. Also, it is unlikely that private property owners compensate those who willingly provide public goods by adding more trees to the property in multifamily buildings [42]. To overcome externalities, the municipal government may promote tree planting and incentivize the property owners [13,68,74]. Urban forests also entail opportunity costs. The trees on the property take up spaces for other potential land uses, such as lawns, garden beds, and swimming pools [66], and urban green spaces limit the parking spaces in auto-oriented cities [53].

Biodiversity from urban forests can trigger insecurity and health risk. The forests and trees are vulnerable to climate change stresses, such as heat, drought, pests, and extreme winds, and therefore could potentially expose the residents to the risk of allergies, fire, earthquake damage, and/or pest invasion [42,73]. Dense forests are considered as disamenity and have a negative influence on house value in western dry-mixed conifer WUI (Wildland Urban Interface) regions of the U.S. because of high fire risk perceptions [50,54] and tree infestation by pest [75]. While there is perceived amenity value in controlled and managed vegetation, such as parks and roadside greenery vacant land plots with trimmed glass, the fear of insecurity and health risk increases if the forests are undomesticated [69]. Belcher and Chisholm [57] reported negative effects of high conservation value vegetation (primary forest, freshwater swamp forest, freshwater marsh, mangrove forest, or old secondary forest) and spontaneous vegetation (scrubland and young secondary forest) on residential property value in Singapore. This is attributable to few recreational facilities in these vegetation types and perceived threats from dangerous or nuisance wildlife (snakes, mosquitoes, macaques, etc.).

Urban forests may also have a gentrifying effect as the increased property value from the investment in tree planting can displace lower-income households. Since urban low-income neighborhoods often have relatively poor access to well-maintained and safe parks, other types of urban open space, and are located in older or industrial areas of cities, they are likely to become the target of urban greening projects with the goal to upscale the neighborhood to become more livable and attractive. In turn, the newly implemented green amenities can have a positive impact on housing prices and neighborhood desirability, which may set off gentrification [28]. The causal link between gentrification and tree planting is not proven, but the case study in Oregon, Portland (USA) found that tree planting drives the increase in the median sales price of single-family housing, which demonstrates that, at the neighborhood level, tree planting and gentrification may be codetermined [76].

## 4 Discussion

### 4.1 Climate zones and concerns

Different types of urban forest management projects (e.g., ecosystem restoration, tree/forest conservation and retention, silviculture, etc.) must take into consideration differences in climate zones and concerns at residential neighborhoods, districts, or county scales. Hotter climate neighborhoods and districts will need more trees and forest cover to increase shade and ameliorate hot temperatures during summer than urban areas existing in cool and cold climates. Thus, tree planting in targeted urban areas should mitigate the growing problem of urban heat islands (UHI) in cities, especially in industrial zones, for climate change mitigation and urban sustainability. This is because urban forests offer high thermal benefits through surface shading and reduction in land surface and air temperatures by intercepting incoming solar radiation. A 1% increase in tree canopy cover could possibly reduce air temperature by 0.14 °C, and an increase in tree canopy cover from 10% to 25% yielded a 2 m air temperature reduction of up to 2.0 °C [77]. Similarly, urban green spaces with trees are more effective in reducing land surface temperatures, with a cooling effect of approximately 2–4 times higher than treeless urban green spaces in summer and during hot extremes [78]. The potential climate change adaptation-related local cooling, carbon sequestration, and pollution reduction effects of urban trees at property, neighborhood, and suburban levels are well documented, among other benefits [1,73,[79], [80], [81]]. Tree inventory in hotter climate zones of California communities, U.S., indicated that there is even ample opportunity to plant more trees to provide more ecosystem services such as climate regulation [73].

The geographical distribution of urban forest ES influences on housing prices, and costs versus benefits of urban forest management are highly limited to countries such as the U.S., China, and those in Europe. These specific neighborhood-level tree cover and city level forest cover needs can be ascertained using LiDAR point cloud data and multispectral imagery to detect and reconstruct individual tree crowns and represent them within 3D city models [82,83]. RS technologies cover extensive spatial areas and provide historical data over a longer period of time, which can be used to conduct large-scale studies across cities to measure urban forests biomass for carbon credits payments, vegetation greenness, ES, detect tree health and forest fragmentation, and monitor urban forest fire. RS techniques can be used to assess urban heat islands and plan urban forests, therefore adding value to residential properties in the area. Although data from UAV was not reportedly used in any study, UAVs offer a viable option for fast, more frequent repeated measurement of individual tree crown detection in urban areas [[45], [46], [47]].

In some cities in the U.S., such as Los Angeles, apps have been developed with access to the public to track tree canopy coverage/density and to determine which neighborhoods need more trees as a means of fighting extreme temperatures (https://phys.org/news/2020-11-los-angeles-google-partner-tree.html). In addition, Texas A&M Forest Service has developed several applications such as the forest carbon clock widget, TreeMD website, trees count mobile app, forest distribution application, urban tree canopy application, etc., respectively, to display real-time data of carbon sequestered and stored in forests, tree health by species and symptoms, map the trees in communities, display the various types of trees statewide and biomass within 50, 75 and 100 miles from a user-defined location, displays predicted urban growth areas and urban tree canopy for selected communities (https://texasforestinfo.tamu.edu/). Other apps (e.g., City Trees (https://apps.apple.com/us/app/city-trees/id1447048394) or tools offered by companies such as CTrees (https://ctrees.org/) can be used to identify and count trees and measure the amount of forest carbon sequestration and storage in city spaces with different weather conditions and/or for different seasons. Five mobile phone apps, namely, NatureID, British Tree Identification, City Trees, Deciduous Trees 2.0 Lite, and Identitree Starter Kit, have been identified as the best apps for identifying trees in urban areas (https://www.makeuseof.com/best-apps-for-identifying-trees/). Data of the individual trees detected by such apps can be used in the housing markets to add value to residential properties of different climatic zones.

### 4.2 Cost-benefit analysis for urban forests

Although urban forest management involving tree planting, maintenance, and protection require continual investment costs of time and money, urban forests provide a wide range of benefits to the population. These potential benefits of urban forests are detailed in Ref. [1], and which include the increase in property values and additional income for landowners through ES credits. Interestingly, the benefits of urban forests to human well-being have been shown to outweigh the costs of urban forest management in monetary terms. For example, an average annual per tree management cost of 19 USD and benefit of 47.83 USD, resulted in a 2.52 USD returned benefit for every USD spent for 173.2 million trees in California, USA [73]. This suggests that the costs of tree planting and management can be captured in residential property values and paid for by house owners and rentals because of the ES benefits urban forests provide to the residents.

Urban forests offer important regulating services such as cooling the built environment through shade, interception, and evapotranspiration, improving local climate and building resilience, store and sequester carbon to mitigate climate change, reduce stormwater runoff, and remove air pollutants [73]. A large urban forest provided a cooling effect of up to 8.4 °C that extended up to 883 m into nearby built-up areas during nighttime compared with urban reference sites [84]. Urban forests have been reported to help reduce or prevent more than 670,000 cases of severe respiratory diseases and thereby save more than 850 lives annually in the U.S [85] through the superior removal efficiency of particulate matter (PM10) and ground-level ozone (O₃) air pollutants during summer [80]. These climate benefits and avoided expenditures in healthcare driven by available urban forests should increase the value of residential properties in such neighborhoods and cities to sustain urban forest management efforts that benefit both low- and high-income families. Using RS data to measure tree and forest level carbon sequestration for modeling climate-smart cities, climate neutral cities, can add value to residential properties by creating a more stable and desirable climate for people which requires less heating and cooling systems, in turn decreasing energy costs for homeowners.

Urban forests have the potential to provide many other health benefits to the population, including the capacity to reduce, prevent and restore physical, mental, psychological, and emotional health issues by providing access to physical activities and viewing and interacting with diverse forest resources. Higher tree crown volumes were associated with lower medication sales for psychological and cardiovascular health issues among residents due to the restorative benefits associated with regular walking, jogging, strolling, or cycling in urban forest environments [86,87]. In addition, a lower prevalence of obesity has been reported for children living in areas with good access to urban green spaces compared to children with limited or no access to urban green spaces [88]. A reduction in clinical depression was also reported among adults engaging in outdoor walks in urban green spaces [89]. Urban forests with higher levels of biodiversity are associated with a lower mortality rate for heart disease and stroke [90]. However, less urban green space was also reported to be associated with higher mental healthcare expenditure through higher antidepressant prescribing or referrals [91]. Estimates suggest that a 10% increase in urban green space in a community can postpone the average onset of health problems in individuals by up to five years [1]. Increases in urban tree cover and green spaces following extensive tree planting and management that benefits human health and well-being - as well as the spatial expansion of urban settlement - can be better monitored and measured using RS technologies more suitable for larger spatial scales than field survey inventories.

### 4.3 Relation to economic prosperity

The reported results clearly indicate that increased public preferences for urban forest types and willingness-to-pay (WTP) for associated natural amenities vary greatly between neighborhoods across a wide metropolitan area and are influenced by a wide range of neighborhood socio-economic factors. For example, homebuyers have positive preferences for urban green/blue spaces, and their WTP for various urban green/blue spaces are higher in neighborhoods with higher income and population density [[92], [93], [94]] and larger home sizes [63]. In contrast, residents in marginalized and poor neighborhoods are less willing to pay to plant and maintain trees and forest cover due mainly to low financial capacity and societal acceptance, such that tree coverage would be a burden to them. Therefore, high-income neighborhoods are more willing to pay for the benefits trees and forest cover provide than low-income neighborhoods [52]. In the case of peri-urban nature, WTP generally increases with income and wealth among households, although an increase in income and education levels will increase WTP more for households at the lower and middle end, respectively, than those at the high end of the WTP distribution [55]. Differences in WTP along multiple value groups were also reported to be due to heterogeneity in the public's preferences for different types and configurations of urban forest structural and functional attributes [95]. Along with offering options to measure the forest characteristics, with RS, studies could also focus on how various other factors associated with income are interrelated (e.g., the amount of nearby agricultural lands that can be translated to food availability, area of wasteland, which can be a surrogate for increased risk to diseases, etc.).

The cost of planting and maintaining an urban forest can be offset by reduced public health costs. Evidence suggests that investment in tree planting on streets to accommodate more biodiversity would be a cost-effective way to reduce mortality related to cardiovascular disease in urban areas [90]. Thus, a positive change in public preference for urban forests is required, particularly for residents in low-income neighborhoods. This will expand the need for tree planting and retention for all to restore and protect urban ecological sustainability and build more resilient cities for a healthy quality of life for the urban population. However, the positive impact of such well-intended green amenities on housing prices and neighborhood desirability associated with tree planting in low-income neighborhoods may lead to gentrification – displacement of current lower-income residents because of the increase in house prices in the area [23,28,76]. Thus, tree planting projects in low-income neighborhoods should protect the neighborhood-specific ecological, social and cultural sustainability to reflect a more informed urban planning practice and place-sensitive urban policy reforms. In this way, the greening of low-income neighborhoods should not significantly increase residential property prices to make sure that such areas are still affordable to low-income earners as much as it is desirable, following the principle of income-based urban forest equity. This equity in urban forest cover between low and high-income neighborhoods can be monitored and measured frequently using RS technologies to ensure that urban greening projects do not lead to gentrification.

### 4.4 Geographical and temporal variation in studies

**Fig. 9:**
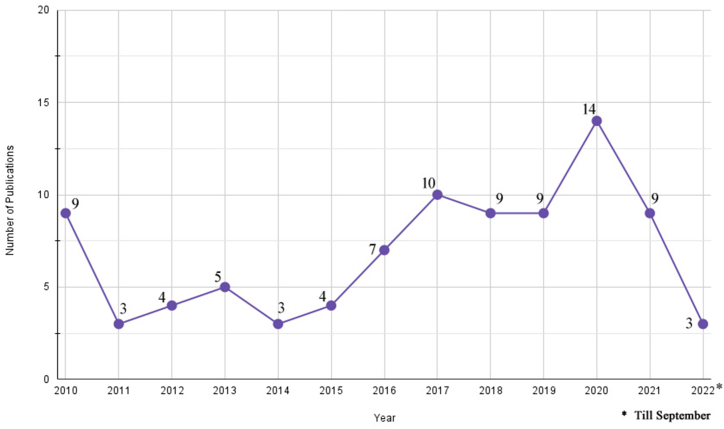
Number of systematic review publications varying between January 2010 and September 2022.

**Fig. 10:**
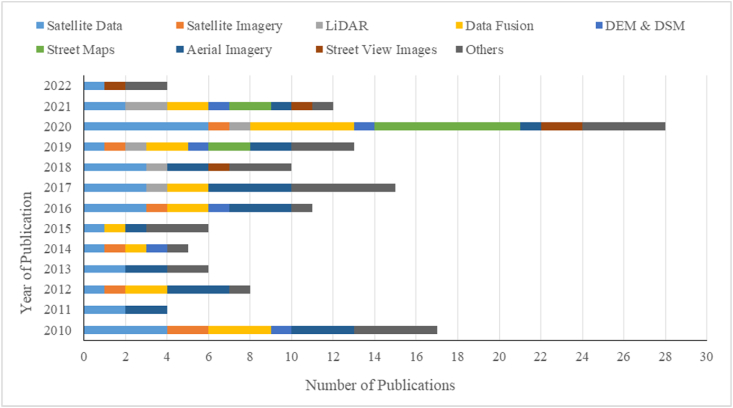
Number of publications and different sources of remotely-sensed data utilized by the studies over time (2010–2022).

Section 3.1 had previously described the country-wise distribution for the 89 studies. To gain further insights, temporal and geographical analysis was performed. Fig. 9 describes the temporal variation of the studies. An approximate annual average of 7 studies on intersecting topics of urban forest, remote sensing and residential property value have been published over the years between January 2010 and September 2022. In regard to remotely sensed data sources (Fig. 10), satellite data and aerial imagery continue to be the most widely used data sources, 30 and 24 papers, respectively. LiDAR started to be utilized in a study in 2017 and appreciably onwards. Moreover, some studies started to apply Street View images and Street maps in 2018, and an increase in their share of the data source each year is notable. The peak year when all the different remotely sensed data sources were used in several of the systematic reviewed papers was 2020.

**Fig. 11:**
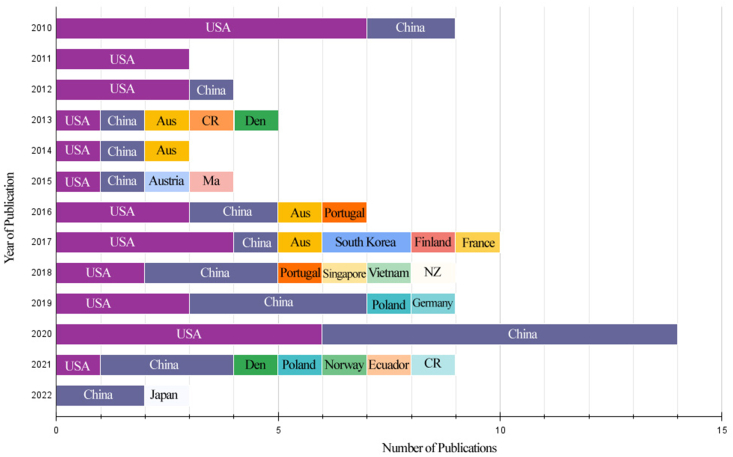
Number of publications for various countries over time (2010–2022). Note (Aus- Australia; Den-Denmark; Por-Portugal; Pol-Poland; SK-South Korea; Fin-Finland; Fra-France; Nor-Norway; Czech-Czech Republic; Ger-Germany; Aust-Austra; Mal-Malaysia; Sing-Singapore; Viet-Vietnam; Ecua-Ecuador; CR-Costa Rica; Jap-Japan; NZ-New Zealand.

We have also observed the geographical focus of studies diversifying over time (Fig. 11). The papers were mostly concentrated on studying urban forests in the US from 2010 through 2012, and later China, collectively leading the country publications through 2022. Except for China, the published studies were focused on the Western world, including the US in particular, and Europe, and Australia. However, from 2018 through 2022, the number of studies published on China surpassed that of the US, Australia and Europe. Besides, studies that focused on urban areas in developing Latin American countries of Ecuador and Costa Rica were only first published in 2021 were published, whereas some studies were earlier published in Malaysia, Vietnam, and Singapore Asia developing countries. No remote sensing-based urban forests and residential property values studies were found for developing countries of Africa in the systematic literature review.

### 4.5 Implication of large players in the market

The real estate developers are key decision makers in shaping cities and the resilient green city orthodoxy. As the orthodoxy persists, the profit margins from greening are attracting real estate developers to locate residential properties near green or greening projects and invest in green properties. According to the [96]'s interviews with residential real estate developers from 15 cities in the US, Western Europe, and Canada, one-quarter of them indicated that urban greening adds economic value and that they perceived green values as a development incentive. They mentioned parks, green spaces, or blue spaces upgrade the design and performance of housing and energize the communities, which in turn, adds value to housing. Residential real estate developers locate their developments adjacent to new or up-and-coming urban greening projects to appropriate surplus value for their properties and receive higher rents, thereby gentrifying neighborhoods for their financial benefits. Greening can reduce risk and validate development as it delivers a positive return for investors. They also claimed that greening is driven by the desire of the consumers to offset the inconvenience of increasingly denser residential development. As the greenways create a sense of connections between and continuity within neighborhoods and green spaces generate new recreational, leisure, and sports opportunities, the density of the residential area is better accepted by the homebuyers. These counterbalance the undesirable elements of the cities, such as gray landscapes, vacant land, and environmental pollutants and toxics [96], and these changes can be effectively tracked and verified using RS data.

Urban greening is mentioned more frequently by the trend-leading private firms, which are other key decision-makers in materializing the urban built environment. For example, ARUP, one of the leading global engineering firms, declared that green infrastructure should play a more significant role in the integrated planning and design of future cities in order to address social, environmental, and economic issues in urban environments. They suggested that green infrastructure can act as a vital carbon sink and provide effective mitigation against risks such as flooding, heatwaves, and drought. It also recommended the exploitation of different layers of the city and vertical space, such as roofs, balconies, and walls, to increase green space as possible. Moreover, they mention the potential of vertical farming as a solution to global food shortages, given it is possible to produce food and harvest energy within limited urban spaces [97]. In this regard, RS possesses the ability to provide near-real-time updates on the progress of certain projects, their impacts, and offer transparency to the general public as well as policy makers.

### 4.6 Future carbon credit prospects

There is a growing trend in the expansion of environmental or ecosystem markets in developed countries where farmers, rangers, and forest landowners receive payments/monetary compensation from different entities for effectively implementing afforestation/reforestation, avoiding conversion and improving forest management projects that reduce CO_2_ in the atmosphere. Carbon markets, a good example of environmental markets, are trading systems where forest landowners voluntarily sell measured, quantified, verified, and tracked carbon credits to entities such as countries, businesses, individuals, or other entities [98,99]. Forest landowners can voluntarily sell carbon credits using a standard carbon unit in metric tons of carbon dioxide equivalent (MtCO_2_e) to known businesses and individuals [99]. Thus, the CO_2_, for example, sequestrated and stored by the different types of urban forests, should be seen as ES carbon credits that could be sold and purchased in the environmental markets while simultaneously holding the potential to be valued in residential property pricing. RS technologies - especially LiDAR and high-resolution imagery (e.g., Planet data) can be used to monitor and measure urban forests aboveground biomass, which is used to estimate the amount of sequestrated carbon and calculate the carbon credits of urban forest types, which can then be reflected in residential property prices. With UAVs, growth status of recently planted trees can also be derived, which could lead to tapping into ex-ante carbon credits [44].

In most improved forest management projects, the business entities will pay the private forest landowners to keep their forests sequestering carbon to offset a portion of carbon emissions from their business operations [98,99]. Such payments can be a viable alternative and additional source of income and economic incentive to motivate private landowners’ participation in the implementation of urban forest management and conservation projects [99,100]. Such entities usually engage in carbon trading to meet internal sustainability goals that demonstrate their commitment to achieving progress in environmental protection and the global sustainable development agenda [99]. Besides, participation in market-based conservation programs encourages landholders to redesign their private land to support improved management for both productive and biodiversity conservation outcomes while attaining specific social outcomes such as social learning and building social capital [101]. Therefore, the creation and expansion of voluntary carbon markets for different urban forest types would motivate private property and forest landowners to reduce and avoid deforestation and support forest management and biodiversity conservation projects, even when urban forest regulations are weak or liberalized. And for scaling these projects there is a need to rely on RS data and by making the measurement process open source, the transparency and credibility of voluntary carbon markets will be enhanced. Carbon credits for urban forests are increasingly gaining widespread public and private interests in the U.S, and increasingly reflected in residential property values. For example, in Seattle, a nonprofit organization called City Forest Credits is the registry that calculates how much carbon is absorbed in smaller urban forestry projects and provides data to earn those credits over the contract period to interested companies such as Regen Network Development (https://www.npr.org/2022/10/04/1126806846/two-groups-want-to-put-focus-on-carbon-credits-from-urban-forests). RS can play a prominent role in estimation related to carbon sequestered by urban forests.

### 4.7 Ecosystem services

Urban forests of all types have the potential to provide a plethora of ES and benefits that should improve the quality of life and well-being of urban dwellers [1]. Urban forests were reported to provide recreational, cultural (aesthetic), and regulating benefits such as reduced home cooling energy use, CO_2_ sequestration and storage, increased rainfall interception, air quality purification in the form of pollution control from GHGs, and stormwater control, and these benefits increased residential property values [73]. Urban forests provide an annual value of ES benefiting urban dwellers that far surpasses the average annual per tree management cost in the US [73]. Improvement of urban ecosystem services was reported to increase housing prices by about 23.67% in 284 cities in China from 2015 to 2018 [102]. With temporal liberalization of urban forests regulations, trees on private properties are massively removed by house owners, leading to the loss of tree canopy cover and the associated ES and structural values benefiting urban residents [103,104]. Thus, a city's forest conservation regulations and strategies should always enforce the retention of trees on private properties and forested conservation lands near residential development, as a potential method for maximizing property and community tree cover [24] to protect and sustain urban forests ES for city residents. These ES generated through forest conservation practices by landowners should be seen as ES credits that can be sold and purchased in the environmental markets, to provide an additional source of income and economic incentive to motivate private landowners' participation in urban forest conservation programs [100].

Most of the reviewed studies focused implicitly and/or explicitly on evaluating how recreation and aesthetic (cultural) services of urban forest types in neighborhoods influenced residential property values. Examples of such studies reported positive effects of resident green viewshed from windows [[60], [61], [62], [63],^105^] and proximity to urban forests types for hiking, biking, jogging, exercising, observing plants and animals [19,24,55,69,106,107] on housing prices. Urban forests also have direct health benefits to residents relating to improvement in psychological, emotional, physical, and mental health of urban residents [1]. Studies that reported positive effects of trees on residential lots and street footpaths on housing prices implicitly suggest that the shade, shelter, air pollutants removal, noise reduction, local climate cooling, and mitigation, etc. provided by these trees are well captured in residential property values by the housing market. The positive economic values and effects of supporting and provisioning services of urban and peri-urban forests on residential property values were not covered in most of the reviewed studies. Evidence suggests that the fusion of field inventory and remotely sensed data provides a viable option to accurately assess forests and tree parameters and ES structural values and losses [104]. Notwithstanding, more studies explicitly providing accurate measures and economic values of urban forests ES using RS technology data for inclusion in hedonic models of residential property pricing are needed to improve our understanding of this important relationship in rapidly changing urban landscapes.

### 4.8 Ecotourism

Ecotourism is responsible and purposeful travel to natural areas that can socially and economically sustain the well-being of the local people while conserving the environment. Ecotourism also creates knowledge and understanding through the interpretation and education of visitors, staff, and host community residents [108,109]. In particular, large urban and peri-urban forests and woodlands, which are managed and at least partly equipped with facilities provide ideal opportunities for ecotourism, leisure, and recreation for urban dwellers [1,110]. Zambrano-Monserrate et al. [10] reported that medium and large urban and peri-urban forests intended for biodiversity conservation are attractive for recreation, leisure and ecotourism activities and have a positive effect on housing prices in Ecuador. However, only very few RS studies have explicitly examined how urban forest ecotourism benefits have been capitalized in residential property values. The conservation and restoration of urban and peri-urban forests for ES and ecotourism benefits to urban dwellers requires careful planning that promotes long term ecosystem diversity and resilience, which can be monitored and measured cheaper, more frequently, over a short time and more extensive and mountainous areas using RS technologies than field survey. Private forest landowners must avoid monocultures while conserving and restoring forests for ecotourism benefits, even though they might be more cost effective, because they are in the long term a hindrance to ecosystem resilience, particularly in events of natural disturbances such as pests, wildfires, and wind.

### 4.9 Changes happened during the COVID pandemic

A total of 26 articles on topics related to the urban forest, remote sensing, and residential property value were published since the outbreak of COVID from January 2020. The average number of articles published in 2020 and 2022 of 8.7 is higher compared to that of pre-COVID (2010–2019) of 6.3. Despite the average increase of 2.4 in the number of papers published between the two periods, trends in an increase in the number of publications were observed from 2016 to 2020, though highly variable; therefore COVID-19's impact on the increase is unclear. However, several studies have been conducted demonstrating the importance of urban green spaces in urban resilience to public health emergencies and resultant change in home amenity preference after the outbreak of COVID-19. According to the article by the Centers for Disease Control and Prevention, exposure to urban green spaces can have a positive effect on the physical and mental health conditions, including reduced levels of obesity, stroke, heart disease, depression, and stress [111]. A study in the US found that urban vegetation led to a 2.6% decrease in cases of COVID-19, suggesting that urban vegetation could mitigate the spread of COVID-19 in communities [112].

As the importance of urban green open spaces in case of crisis and extreme situations are revealed through the experience of COVID-19, access to large-scale, high-quality urban green spaces that enable residents to walk and cycle while adhering to social distancing are considered a valued urban asset in dense urban settlements [113]. Accordingly, the recent studies indicated apparent trends in increased preferences of residents and investors for housing close to urban green spaces. In the case of Poland, a survey of preferences regarding contact with nature in the place of residence in Polish cities revealed an increased preference for urban green areas in the context of the COVID-19 pandemic. While only 11.16% of respondents considered proximity to urban green space important prior to the pandemic, 55.7% of respondents considered it important during the pandemic [114]. Investors in Poland also have shifted their housing preference to areas located near forests and lakes. A study of real estate transactions in Polish cities between 2018 and 2020 informs that as remote working conditions become more common and the location of the property holds less importance, investors focus more on specific characteristics of the house, such as access to green space, and the possibility of having one's own garden [115]. Besides, using RS data to study urban forests and residential property values relations was more practical and feasible during COVID-19 restrictions than engaging in field surveys to obtain data on urban forest and tree parameters.

### 4.10 Limitations to exploiting urban forests-residential property synergies

In general, the lack of accurate measures and understanding of the valuation of most variables of individual ES - such as regulating services (e.g., regulating air quality, microclimate, water quality, and noise) and supporting services (e.g., nutrient cycling, soil development), and difficulty of finding a hedonic pricing model specification of different ES, limits their valuation in residential property pricing [3]. There are limited numerical models available to quantify a wider variety of ES [73], evidenced by the limited number of ES that are evaluated in i-Tree Eco software [104], for potential inclusion in hedonic property pricing models. There are also challenges involved in estimating net benefits associated with the costs of managing and preserving urban forest types, including maintenance costs from litter generation, damages to homes caused by windstorms, and replanting after destruction [6], where RS applications could be employed extensively.

In addition, there are shortcomings in the applicability of urban forests-residential property values relations, because residents’ preferences for tree cover and individual trees and forest structure in residential lots and neighborhoods, respectively, may change over time [24]. For example, private property owners' preference for trees decreased significantly with liberalization of urban forests regulations in Poland, leading to massive loss of tree canopy cover and associated ES and structural values [103,104]. For developing countries in particular, there is widespread limited or no data and/or lack of access to large scale detailed spatial, housing rental, and socio-economic data from governments and real estate companies [9]. This indicates why there are limited studies providing evidence of the effect of urban forests on housing prices in developing countries [10]. Given the availability of new kinds of open-source high-resolution global scale RS data (such as NASA GEDI spaceborne LiDAR), further research for large-scale urban forests should be prioritized.

Urban forest types and their benefits to human well-being and the related side-effect on housing prices were found to be more valued in developed countries compared to developing countries. Trees in residential lots (particularly in low-income neighborhoods) [16,52], and distant unmanaged forests in developing countries [10,57,70] were reportedly perceived as disamenity and devalued housing prices because of the dangers they pose to residents. Conversely, only in two studies were neighborhood forests perceived as disamenity in developed countries [13,68]. This suggests the need for transforming the mindsets of residents and urban forestry policy reforms in both developing and developed countries based on city-urban area-specific requirements of forests and tree canopy cover. This will help achieve a win-win urban land-use planning and management against targeted urban forestry-related SDGs. In developing countries, more awareness campaigns should be initiated to motivate locals to participate in pro-environmental activities involving the planting, caring and preserving of native trees and green vegetation in urban landscapes [10], along with learning the usage of open-source RS data for canopy assessment, mapping and quantification.

### 4.11 Low-cost Unmanned Aerial Vehicles (UAVs) as an assessment tool

As mentioned in *3.3,* satellite and aerial imageries are the dominant remotely-sensed data sources for the previous studies in mapping the tree canopy cover. Even though the usage of satellite imagery is fast and objective, it is difficult for satellite imageries to maintain good quality simultaneously with spatio-temporal resolution and three-dimensional information [105,116] and to provide details of complex and fragmented urban forests [106]. Some studies have pointed out that its overhead view provides less connection with residents’ intuitive visual perceptions [106,117]. Among our reviewed studies, some studies since 2018 have incorporated street view images to overcome the limitation of satellite imageries to reconstruct the horizontal view perspective of residents [36,61,63,105,117]. However, it is not without limitations, as it is difficult to find data with specific acquisition times as urban areas go through rapid development and seasonal changes [105].

UAVs imagery offers high spatial resolution data [44,116,118] in a cost effective and time efficient manner [118]. Isibue and Pingel [118] used UAV data to produce estimates of tree height in the urban environment and concluded that the results from UAVs is comparable to manual field measurement [118] and even better than conventional aerial LiDAR [116,118]. In addition, it provided improved accuracy in younger, smaller trees [118], leaf characteristics, canopy diameter, single tree segmentation, and tree classification [116] that are often not well-reflected with LiDAR.

UAVs have been employed to map and update real estate in rapidly developing areas to deliver cadastral and community maps and real estate ownership surveys. An experimental study was conducted to extract cadastral boundaries in Busogo, Rwanda by utilizing UAV imageries and suggested that the data when combined with deep learning models can reduce processing time and labor force compared to the current practice [119]. A World Bank's project in Albania used UAV to improve governance through feasible, manageable, and efficient means to improve spatial data in cadastral and community mapping [120]. A study on the comparison of surveying and mapping methods for housing and real estate ownership confirmation in China estimated that increased work efficiency by 60% and reduced labor cost by 62.5% is expected when using UAV oblique photography compared with the conventional survey practice [121].

### 4.12 Support for United Nations sustainable development goals

Urban development will affect forests near and far from urban centers. The adverse effect of urbanization on urban forests and associated ecosystem services can be minimized with the active inclusion of urban and peri-urban forest in city agendas and planning. Cities have been prioritizing provision of basic services but have neglected encouraging inclusive access to urban forest. Synergizing UN SDGs would benefit both the urban dwellers and forest communities [122]. Urban forests directly and indirectly contribute to the achievement of the UN SDGs. Urban forests offer many economic benefits and boost green economies which can raise the quality of life of low-income urban residents and lift them from poverty (SDG1). They contribute to food security and nutrition directly by supplying nutritious foods such as fruits and seeds and indirectly by supporting agricultural production by providing wood fuels, high-quality water, and improved soil (SDG2). The forests and other green spaces prevent diseases by removing sources of non-communicable diseases (pollutants and particulates) and urban stressors (ultraviolet radiation and noise pollution) and creating recreation amenities with therapeutic benefits for mental health (SDG3) [1]. With RS, urban planners would be able to easily locate and/or prioritize revegetation zones at city scales.

In addition, urban forests enhance the availability and quality of water by filtering the pollutants in water and easing the risk of damaging runoff and urban flooding (SDG6). As wood fuel is renewable bio-based, and often the most affordable energy source, urban forests can generate power and heat for low-income urban households without consuming fossil fuel and increasing the pressure on natural forests (SDG7). Investment in urban forests can sustainably create employment, increase income, provide a resource for entrepreneurs, cut the urban infrastructure cost, supply ecosystem services, improve the living environment, and increase property values (SDG8). Urban forests play an incremental role in making cities livable, environmentally sustainable, and economically viable (SDG11). The trees and forests directly mitigate climate change as they sequester carbon and reduce greenhouse gas emissions and indirectly save energy and alleviate the urban heat island effects (SDG13) and advanced RS techniques can be utilized to track the global carbon balance. Urban forests also conserve natural resources and biodiversity and are habitats for urban wildlife (SDG15) [1], which can in fact be modeled using RS data (e.g., LiDAR) effectively.

### 4.13 Limitations of the study

The study did not investigate the effectiveness of the different remote sensing data sources in accurately and efficiently measuring urban forests and tree-level characteristics and how this improved on the accuracy of estimating the influence of urban forests types/characteristics on residential property values in hedonic models due to limitations in data availability in the reviewed studies. The study considered only papers on remote sensing-based urban forests and residential property values relations published from 2010 to 2022 in three main databases (WOS, Scopus, and Google scholar); this may have caused the missing out of some relevant literature published before 2010 and spread in other databases.

### 4.14 Potential future research opportunities and policy implications

Urban forests will continue to have a tremendous influence on residential property values, especially in developed countries where real estate stakeholders are increasingly capturing urban forests ES amenity benefits in housing prices. However, the current status and trend of this relationship is largely unknown in developing countries due to lack of studies. There exists a great disparity in knowledge on the relationship of urban forests and residential property values between developed and developing countries, with a highly limited number of remote sensing-based studies in the latter compared to the former.

More studies were conducted for tree canopy cover, and the number of trees in residential lots and proximity to nearest neighborhood managed urban forests and green spaces compared to large-scale distant unmanaged and developed urban forests and peri-urban forests greater than 0.5 ha with more diverse ES amenity benefits. In general, there was a paucity of studies that focused on individual tree parameters, such as tree species, tree size (DBH), tree height, tree condition, tree shape, tree age, tree condition, and forest types (deciduous and evergreen broadleaves, conifers, tropical dry and rainforest forests, etc.) and their effect on housing prices.

There is an urgent need for more studies around the globe in this aspect - especially in the Global South and developing countries in particular, where there is a paucity of studies and limited evidence of the relationship between interactive urban forest variables and residential property values. Urban green spaces in cities in developing countries are valued differently between house owners and house rentals; and because they are mostly poorly managed, they tend to devalue housing prices, as they constitute negative externalities to the people in a city [9,10]. Single family house owners assign substantial value to large and proximate urban green spaces such as neighborhood parks than house rentals [9]. Interestingly, there is increasing value and more willing to pay for the restoration of non-developed and degraded urban green areas to benefit from urban ecosystem services in cities in developing countries [[17], [18], [19]]. Thus, the regeneration of urban green spaces in cities in developing countries might have substantial positive impacts on the dynamics of housing prices across different categories of housing markets in the near future, especially for single family houses. Thus, it is important to study the role of urban forests on real estate properties in cities in developing countries, where urbanization is mostly rapid, unregulated and uncontrolled, to further understand this dynamic relationship and help inform urban planning policy and practice towards the protection, conservation and construction of urban green spaces.

UAVs are more convenient to use for individual tree detection for pocket parks and gardens with trees, trees on streets or in public squares and residential lots, and other green space urban forest types. In addition, tree parameters such as tree canopy cover and number of trees can be manually mapped over long-term periods using high-resolution remote sensing images from Google Earth as a low-budget solution compared to field surveys, especially when the geographical area is small or sampled areas are used as in a sampling-based approach, instead of the large geographical area [103]. Furthermore, it is feasible to combine high-resolution LiDAR data in conjunction with historical aerial photographs to detect tree canopy cover changes over longer time scales and over small urban forest areas when inconsistent data types are available between the two time periods [104,123]. Overall, we need to adopt and apply technological advancements made in the fields of precision forestry, data science, and remote sensing to improve our further understanding of the influence of urban forests and residential property values [43]. Below we provide some specific recommendations for future research based on our review of the literature.

● Applications of advanced RS technologies that can accurately and efficiently measure individual items of urban ES categories and tree-level characteristics to enable their appropriate valuation in residential property pricing, and how these can be included in specific and/or integrated property pricing in hedonic models.
● Modeling studies to understand public preferences for future changes in tree cover and individual trees and forest structure on both residential lots and neighborhood scale in order to match future demand and supply of urban forests-residential property values relations in rapidly changing urban landscapes.
● Mathematical models that seek to simulate the effectiveness of creating carbon credit markets for urban forests to expand residential property and urban forest landowners' income as a strategy to promote urban forest retention and reforestation in different geographical and economic contexts.
● Novel remote sensing-based studies estimating urban forest greenness using different combinations of vegetation indices for the different urban forest types for optimal results.
● Next-generation models - with multiple counterparts - where we can add in multifarious data, including near real-time remote sensing data pertaining to various environmental variables and socioeconomic situations.
● New satellite imagery-based studies targeted at understanding how much percentage of new real estate residential property developments were assigned for urban forest cover in developing countries in the past decade, particularly those of Africa; and how is this trend changing over time; how is this different for cities (with limited space for expansion) vs suburbs. More studies on urban forests and residential property values relationship using remote sensing technologies are generally needed for developing countries of Africa in particular, due to observed paucity of studies in this literature review.
● Future remote sensing studies estimating urban forest matrices such as tree canopy cover over longer periods of time at small spatial scales and residential property relations should combine historical and modern remote sensing technology data types as a way to complement long-term data limitations of modern remote sensing tools such as LiDAR and UAVs.
● Region-wide LiDAR data collection campaigns for provision of long-term data for estimating urban forests and vegetation characteristics to guide urban planning and development policy reform and practices applicable to the housing market sector.

## 5 Outlook and concluding remarks

Remote sensing techniques have shown great prominence and potential to measure and provide data on urban forest parameters, especially at larger spatial scales and over longer time periods, to help improve our understanding of urban forest influence on residential property values. We reviewed 89 studies from January 2010 to September 2022 that used various RS technology data to quantify different vegetation, forest, and tree parameters in urban forests and estimated their effects on housing prices. The majority of the studies used a combination of various regression estimation methods in hedonic models to estimate the urban forest effect on residential property values. However, ES valuation models showed some potential and can be comprehensively used as more numerical model software accommodating multiple individual ES variables are developed. The paucity of urban forest-residential property values-RS studies in developing countries, particularly in Africa, highlights a long-standing geographical distribution disparity context in such studies between developed and developing countries. Tree canopy cover and the number of trees on the property, in addition to forest size, vegetation type, greenness, and proximity to neighborhood and peri-urban forests at different spatial scales, were the primarily used parameters, with mostly positive effects on housing prices.

Urban forest cultural services such as recreation and aesthetics, and regulating services such as air quality purification and cooling local climate were the primary benefits mostly found to increase housing prices in the reviewed RS studies. The explicit estimation of the economic value of some urban forest benefits, particularly individual regulating and supporting services, remains a big challenge, mainly due to a limited understanding of these ES and their valuation. RS techniques with high-resolution data such as satellite and aerial imagery, street maps, and LiDAR data offer superior efficiency to quantify vegetation, forest, and tree parameters for different urban forest types compared to field inventory data. Future RS studies on urban forests and residential property values should also explicitly measure and quantify other important urban forest parameters such as tree species, tree size, tree shape, tree condition, tree age, tree height, tree density, LAI, vegetation greenness using NDVI, and various ecosystem services for inclusion in hedonic models. Because increased urban forest presence at desired thresholds in properties, neighborhoods and suburbs mostly increases housing prices, and the benefits outweigh the costs, private landowners who participate in urban forest reforestation and conservation activities can earn additional income from ES credits. Therefore, promoting reforestation, conservation and monitoring of different urban forest types using remote sensing data and techniques should be encouraged, to ensure urban sustainability and resilience, since urban forests have the potential to contribute to the achievement of nine UN SDGs. The insights and recommendations shared in the paper are expected to aid urban policymakers, governmental and non-governmental institutions, property developers, researchers and other audiences with regard to the current status and application of remote sensing in comprehending influences of urban forests on residential property values.

## Data availability statement

Data will be made available on request.

## Funding

This research received no external funding.

## CRediT authorship contribution statement

**Ewane Basil Ewane:** Conceptualization, Data curation, Formal analysis, Investigation, Methodology, Project administration, Visualization, Writing – original draft, Writing – review & editing, Supervision, Validation. **Shaurya Bajaj:** Conceptualization, Data curation, Formal analysis, Investigation, Methodology, Project administration, Visualization, Writing – original draft, Writing – review & editing, Supervision, Validation. **Luisa Velasquez-Camacho:** Data curation, Formal analysis, Methodology, Validation, Visualization, Writing – original draft, Writing – review & editing. **Shruthi Srinivasan:** Data curation, Formal analysis, Methodology, Writing – original draft, Writing – review & editing. **Juyeon Maeng:** Data curation, Formal analysis, Methodology, Writing – original draft. **Anushka Singla:** Data curation, Formal analysis, Methodology, Visualization, Writing – original draft. **Andrea Luber:** Data curation, Formal analysis, Methodology, Writing – original draft, Writing – review & editing. **Sergio de-Miguel:** Formal analysis, Methodology, Writing – original draft, Writing – review & editing. **Gabriella Richardson:** Formal analysis, Methodology, Writing – original draft, Writing – review & editing. **Eben North Broadbent:** Formal analysis, Methodology, Writing – original draft, Writing – review & editing. **Adrian Cardil:** Formal analysis, Methodology, Writing – original draft, Writing – review & editing. **Wan Shafrina Wan Mohd Jaafar:** Formal analysis, Methodology, Writing – original draft, Writing – review & editing. **Meshal Abdullah:** Formal analysis, Methodology, Writing – original draft, Writing – review & editing. **Ana Paula Dalla Corte:** Formal analysis, Methodology, Writing – original draft, Writing – review & editing. **Carlos Alberto Silva:** Formal analysis, Methodology, Writing – original draft, Writing – review & editing. **Willie Doaemo:** Formal analysis, Methodology, Writing – original draft, Writing – review & editing. **Midhun Mohan:** Conceptualization, Data curation, Formal analysis, Investigation, Methodology, Project administration, Resources, Supervision, Validation, Visualization, Writing – original draft, Writing – review & editing.

## Declaration of competing interest

The authors declare that they have no known competing financial interests or personal relationships that could have appeared to influence the work reported in this paper.
