# Supplementary material for: Influence of urban forests on residential property values: A systematic review of remote sensing-based studies
Source: Heliyon. 2023 Sep 24;9(10):e20408. doi: 10.1016/j.heliyon.2023.e20408 (PMC10568372; doi:10.1016/j.heliyon.2023.e20408)
Supplement: Multimedia component 1 [file mmc1.docx]

**SUPPLEMENTARY MATERIALS**

Supplementary Material Table S1. Summary of the journals where the reviewed papers on remote sensing-based urban forests and residential property values relations were published

| **SN** | **Journal** | **Number of papers** |
| --- | --- | --- |
| 1 | Landscape and Urban Planning | 12 |
| 2 | Urban Forestry and Urban Greening | 11 |
| 3 | Sustainability | 9 |
| 4 | Ecological Economics | 6 |
| 5 | Forest Policy and Economics | 5 |
| 6 | Land Use Policy | 3 |
| 7 | Cities | 3 |
| 8 | Habitat International | 3 |
| 9 | International Journal of Geo-Information | 2 |
| 10 | Regional Science and Urban Economics | 2 |
| 11 | Ecological Indicators | 2 |
| 12 | Applied Geography | 2 |
| 13 | International Journal of Environmental Research and Public Health | 2 |
| 14 | Journal of Urban Planning and Development | 2 |
| 15 | WOS | 2 |
| 16 | Computers, Environment and Urban Systems | 1 |
| 17 | Journal of Environmental Planning and Management | 1 |
| 18 | Journal of Resources and Ecology | 1 |
| 19 | The International of Real Estate Finance and Economics | 1 |
| 20 | Arboriculture and Urban Forestry | 1 |
| 21 | Urban Ecosystems | 1 |
| 22 | PLOS ONE | 1 |
| 23 | The Annals of Regional Science | 1 |
| 24 | Land | 1 |
| 25 | Land Economics | 1 |
| 26 | Environmental Planning B. | 1 |
| 27 | Ecosystem Services | 1 |
| 28 | Procedia-Social and Behavioural Sciences | 1 |
| 29 | International Journal of Construction Management | 1 |
| 30 | Urban Studies | 1 |
| 31 | Forests | 1 |
| 32 | Applied Economics | 1 |
| 33 | Journal of Forest Economics | 1 |
| 34 | Remote Sensing | 1 |
| 35 | Southern Journal of Applied Forestry | 1 |
| 36 | Australian Journal of Agricultural and Resource Economics | 1 |
| 37 | Forest Science | 1 |
| 38 | Journal of Economic Geography | 1 |
|  | TOTAL | 89 |

Supplementary Material Table S2. Summary of the reviewed results on the impact of urban forests on residential property prices using hedonic models based on some remote sensing studies at the neighborhood scale

| **Study** | **Forest characteristics measured** | **Scale of measurement** | **Results** |
| --- | --- | --- | --- |
| Bridges et al. (2020), Chen et al. (2020), Conway et al. (2010), Donovan et al. (2019), Donovan and Butry, (2010; 2011), Hjerpe et al. (2016), Li et al. (2015), Li and Saphores (2012), Netusil et al. (2010), Pandit et al. (2014), Sander et al. (2010), Saphores and Li (2012), Wu et al. (2017),Yu et al. (2020) | Tree canopy cover and green spaces + accessibility/proximity/distance factor | Neighborhood within a radius buffer of 20m to 400m of property | Positive effect of large tree canopy coverage /proximity to green spaces in neighborhood public spaces on housing prices |
| Bridges et al. (2020), Hjerpe et al. (2016), Jensen et al. (2021), Kim et al. (2016), Liu et al. (2020), Park and Kim (2017) | Tree canopy cover and size of green spaces + proximity/distance factor | Neighborhood within a radius buffer of 500m to 800m of property | Positive effect of large tree canopy coverage /green spaces in neighborhood pubic space on housing prices  Positive effect of proximity to large forest size on housing prices |
| Belcher and Chisholm (2018), Bridges et al. (2020), Franco and Macdonald (2016, 2018), Melichar and Kaprova (2013), Price et al. (2010), Wu et al. (2017), Zhu et al. (2019) | Tree canopy cover and green spaces + proximity/distance factor | Neighborhood within a radius buffer of 1km to 3km of property | Positive effect of large managed forest/ tree canopy coverage/ green spaces in neighborhood pubic space on housing prices |
| Li et al. (2016), Li et al. (2019; 2020), McPherson et al. (2017), Tran et al. (2020), Mei et al. (2018a; 2018b), Ma et al. (2022), Wang et al. (2013; 2016; 2020a and 2020b) | Tree canopy cover, vegetation type, area/size of green space, spatial placement of green infrastructure, vegetation greenness (NDVI) + proximity/ distance factor | Neighborhood green vegetation within 135m scale radius buffer | Positive effect of neighborhood greenness (NDVI) on housing prices due to community benefits of ecosystem services |
| Donovan et al. (2021), Pandit et al. (2013), Plant et al. (2017), Plant et al. (2016) | Tree canopy cover, number of trees + proximity/distance factor | Neighborhood street footpath within 100 of property | Positive effect of street footpath tree canopy cover on property prices |
| Conway et al. (2010), Hiebert and Allen (2019), Łaszkiewicz et al. (2022), Noor et al. (2015), Sander et al. (2010) | Tree cover, forest size, green spaces + proximity/distance factor | Neighborhood distance within 60m to 400m of property | Positive of proximity to tree cover, large forest and small, medium and large green spaces on housing prices |
| Mei t al. (2018), Zambrano-Monserrate et al. (2021) | Forest size + proximity/distance factor | Neighborhood | Positive effect of small and medium scale forests and green spaces on housing prices |
| Franco and Macdonald (2018), Zambrano-Monserrate et al. (2021) | Forest size, green spaces + proximity/ distance factor | Neighborhood urban forest and peri-urban areas | Positive effect of large urban forest and green spaces in neighborhood on housing prices |
| Chen et al. (2020), Gu et al. (2021), Hiebert and Allen (2019), Zhang and Dong (2018), Zhang et al. (2021), Luo et al. (2022) | Green view + proximity/distance factor | Neighborhood within a radius buffer of 400 m | Positive effect of residents’ high horizontal view of green vegetation on housing prices |
| Belcher and Chisholm (2018), Chwiałkowski and Zydro (2021), Tuffery (2017), Votsis (2017) | Managed forests + proximity/distance factor | Neighborhood distance up to 9km maximum | Positive effect of proximity to large managed forests and distant unprotected regional forest on housing prices |
| Liu et al. (2020), Li et al. (2015), Saphores and Li (2012) | Grass cover + proximity/distance factor | Neighborhood within 800m distance | Positive effect of proximity to nearest moderate irrigated grassland on housing prices |
| Mei et al. (2018) | Urban green vegetation (NDVI) | Neighborhood at radius buffer scale > 135 m | Negative effect of urban green vegetation (NDVI) on housing price |
| Conway et al. (2010), Tuffery (2017) | Forest size and green spaces + proximity/ distance factor | Neighborhood within 90m to 160m radius buffer | Negative effect of proximity to small nearby peri-urban forest and green spaces on housing prices |
| Sander et al. (2010) | Tree canopy cover + proximity/distance factor | Neighborhood 500m to 1000m | Negative effect of tree canopy coverage in neighborhood pubic space on housing prices |
| Wu et al. (2017) | Green spaces + accessibility factor | Neighborhood within 5.7 km distance | Negative effect of accessibility to distant forest parks on housing prices to limited transportation and convenience |
| Jiao et al. (2021) | Tree density and number of trees | Neighborhood within 78,360 m^2^ radius buffer | Negative effect of tree density in neighborhood pubic space on housing prices |
| Ma & Gopal, 2018) | Unprotected forests + distance factor | Town scale | Non-significant effect of proximity to unprotected forests on housing prices |
| Jo-Black and Richards (2020), Chen et al. (2020a; 2020b), Liu et al. (2019), Jim and Chen (2010), Panduro and Veie (2013), Qui et al. (2020) | Urban green spaces + proximity/distance factor | Neighborhood within a radius buffer of 20m to 400m of property | Positive effect of proximity to green spaces within neighborhood on housing prices |
| Cho et al. (2014), Fu et al. (2019, Jiao and Liu (2010), Jim and Chen (2010), Liu et al. (2020), Park et al. (2017), Tam et al. (2019), Tian et al. (2017), Wen et al. (2015) | Urban green spaces + proximity/distance factor | Neighborhood within a radius buffer of 500m to 800m of property | Positive effect of proximity to green spaces within neighborhood on housing prices |
| Biao et al. (2012), Chen et al. (2022), Liang et al. (2018), Liebet et al. (2019), Wu et al. (2014) | Urban green spaces + proximity/distance factor | Neighborhood within a radius buffer of >800m to 2000m of property | Positive effect of proximity to green spaces within neighborhood on housing prices |
| Cho et al. (2010; 2011), Chung et al. (2018), Herath et al. (2015), Poudyal et al. (2010), Xiao et al. (2016) | Urban green spaces + proximity/distance factor | Neighborhood within a radius buffer of >2000m of property | Positive effect of proximity to green spaces within neighborhood on housing prices |
| Hover et al. (2020), Jun and Kim (2017), Łaszkiewicz et al. (2019) | Urban green spaces + proximity/distance factor | Neighborhood within a radius buffer of >500m to 1000m of property | Negative effect of proximity to green spaces within neighborhood on housing prices |
| Holmes et al. (2011) | Tree infestation | Neighborhood | Negative effect of proximity of hemlock woolly Adelgid in residential forests within neighborhood on housing prices |
| Piaggio (2021) | Size of urban green space + proximity/ distance factor | Neighborhood | Positive effect of urban green space and proximity on residential property values for homeowners.  Negative effect of urban green space and proximity on residential property values for home rentals. |

Supplementary Material Table S3. Summary of the reviewed results on the impact of urban forests on residential property prices using hedonic models based on some remote sensing studies at the neighborhood at the housing property/parcel scale

| **Study** | **Forest characteristics measured** | **Spatial scale of measurement** | **Results** |
| --- | --- | --- | --- |
| Donovan and Butry (2010, 2011), Mei et al. (2018), Mei et al. (2017), Kadish and Netusil (2012), Larson and Perrings (2013), Seo (2020), Farmer et al. (2011) | Tree canopy cover and number of trees | Residential lot | Positive effect of tree canopy cover within a residential lot on property values, particularly for middle to high income homes with large lots |
| Bridges et al. (2020), Mei et al. (2018), Mei et al. (2017), Netusil et al. (2010), Pandit et al. (2013), Plant et al. (2016), Saphores and Li (2012), Seo (2020) | Tree canopy cover, number of trees, tree species, tree size and tree condition | Residential lot | Negative effect of tree canopy cover within a residential lot on housing prices (particularly in low income neighborhoods) |
| Li et al. (2015), Li and Saphores (2012), Pandit et al. (2014) | Tree canopy cover and green vegetation | Residential lot | Non-significant effect of tree canopy cover within a residential lot on property prices |
| Li et al. (2015), Saphores and Li (2012) | Grass cover + irrigation factor | Residential lot | Positive effect of parcel irrigated grass cover on housing prices |
| Saphores and Li (2012) | Grass cover + irrigation factor | Residential lot | Negative effect of parcel non-irrigated grass cover on housing prices |

**References (Included in the systematic literature review)**

1. Belcher, R. N., & Chisholm, R. A. (2018). Tropical vegetation and residential property value: A hedonic pricing analysis in Singapore. Ecological Economics, 149, 149–159. https://doi.org/10.1016/j.ecolecon.2018.03.012.

2. Biao, Z., Gaodi, X., Bin, X., & Canqiang, Z. (2012). The effects of public green spaces on residential property value in Beijing. Journal of Resources and Ecology, 3(3), 243–252. https://doi.org/10.5814/j.issn.1674-764x.2012.03.007.

3. Bridges, L.E., Grado, S.C., Gordon, J.S., Grebner, D.L., Kushla, J.D. (2020). The influence of canopy cover on property values in a small southern US City. Arboriculture & Urban Forestry, 46(4):262–275.

4. Chen, S., Zhang, L., Huang, Y., Wilson B., Mosey, G., Brian Deal, B. (2022). Spatial impacts of multimodal accessibility to green spaces on housing price in Cook County, Illinois. Urban Forestry & Urban Greening, 67, 127370. https://doi.org/10.1016/j.ufug.2021.127370.

5. Chen, L., Yao, X., Liu, Y., Zhu, Y., Chen, W., Zhao, X., & Chi, T. (2020a). Measuring impacts of urban environmental elements on housing prices based on multisource data – A Case Study of Shanghai, China. ISPRS International Journal of Geo-Information, 9(2), 106. https://doi.org/10.3390/ijgi9020106.

6. Chen, Y., Yue, W., & La Rosa, D. (2020b). Which communities have better accessibility to green space? An investigation into environmental inequality using big data. Landscape and Urban Planning, 204, 103919. doi:10.1016/j.landurbplan.2020.103919.

7. Cho, S.-H., Kim, T., Roberts, R. K., Hellwinckel, C., Kim, S. G., & Wilson, B. (2014). Developing an amenity value calculator for urban forest landscapes. Computers, Environment and Urban Systems, 43, 34-41. https://doi.org/10.1016/j.compenvurbsys.2013.

8. Cho, S.-H., Kim, S. G., & Roberts, R. K. (2011). Values of environmental landscape amenities during the 2000–2006 real estate boom and subsequent 2008 recession. Journal of Environmental Planning and Management, 54(1), 71–91. https://doi.org/10.1080/09640568.2010.502760.

9. Cho, S.-H., Lambert, D. M., Roberts, R. K., & Kim, S. G. (2010). Moderating urban sprawl: is there a balance between shared open space and housing parcel size? Journal of Economic Geography, 10(5), 763–783. https://doi.org/10.1093/jeg/lbp048.

10. Chwiałkowski, C.; Zydroń, A. (2021) Socio-economic and spatial characteristics of Wielkopolski national park: application of the hedonic pricing method. Sustainability 13, 5001. https://doi.org/10.3390/su13095001.

11. Chung, Y., Seo, D., & Kim, J. (2018). Price determinants and GIS analysis of the housing market in Vietnam: The Cases of Ho Chi Minh City and Hanoi. Sustainability, 10(12), 4720. https://doi.org/10.3390/su10124720.

12. Conway, D., Li, C. Q., Wolch, J., Kahle, C., & Jerrett, M. (2010). A spatial autocorrelation approach for examining the effects of urban greenspace on residential property values. The Journal of Real Estate Finance and Economics, 41(2), 150–169. https://doi.org/10.1007/s11146-008-9159-6.

13. Donovan, G. H., Prestemon, J. P., Butry, D. T., Kaminski, A. R., & Monleon, V. J. (2021). The politics of urban trees: Tree planting is associated with gentrification in Portland, Oregon. Forest Policy and Economics, 124, 102387. https://doi.org/10.1016/j.forpol.2020.102387.

14. Donovan, G. H., Landry, S., & Winter, C. (2019). Urban trees, house price, and redevelopment pressure in Tampa, Florida. Urban Forestry & Urban Greening, 38, 330–336. https://doi.org/10.1016/j.ufug.2019.01.014.

15. Donovan, G. H., & Butry, D. T. (2011). The effect of urban trees on the rental price of single-family homes in Portland, Oregon. Urban Forestry & Urban Greening, 10(3), 163-168. https://doi.org/10.1016/j.ufug.2011.05.007.

16. Donovan, G. H., & Butry, D. T. (2010). Trees in the city: Valuing street trees in Portland, Oregon. Landscape and Urban Planning, 94(2), 77–83. https://doi.org/10.1016/j.landurbplan.2009.07.019.

17. Farmer, M. C., Wallace, M. C., & Shiroya, M. (2011). Bird diversity indicates ecological value in urban home prices. Urban Ecosystems, 16(1), 131–144. https://doi.org/10.1007/s11252-011-0209-0.

18. Franco, S.F., & Macdonald, J.L. (2018). Measurement and valuation of urban greenness: Remote sensing and hedonic applications to Lisbon, Portugal. Regional Science and Urban Economics, 72, 156–180. https://doi.org/10.1016/j.regsciurbeco.2017.03.002.

19. Franco, S.F., & Macdonald, J.L. (2016). Tree canopies, urban green amenities and the residential real estate market: Remote sensing and spatial hedonic applications to Lisbon, Portugal. 11th Meeting of the Urban Economics Association. https://urbaneconomics.org/meetings/uea2016/program.html.

20. Fu, X., Jia, T., Zhang, X., Li, S., & Zhang, Y. (2019). Do street-level scene perceptions affect housing prices in Chinese megacities? An analysis using open access datasets and deep learning. PLOS ONE, 14(5), e0217505. https://doi.org/10.1371/journal.pone.0217505.

21. Gu, J., Wang, X., & Liu, G. (2021). Rediscovering the amenity value of urban landscapes in the mountainous areas with high-rise buildings from the perspective of 3D vertical urban systems. Urban Forestry & Urban Greening, 60, 127018. https://doi.org/10.1016/j.ufug.2021.127018.

22. Herath, S., Choumert, J., & Maier, G. (2015). The value of the greenbelt in Vienna: a spatial hedonic analysis. The Annals of Regional Science, 54(2), 349–374. https://doi.org/10.1007/s00168-015-0657-1.

23. Hiebert, J., & Allen, K. (2019). Valuing environmental amenities across space: A geographically weighted regression of housing preferences in Greenville County, SC. Land, 8(10). https://doi.org/10.3390/land8100147.

24. Hjerpe, E., Kim, Y.-S., & Dunn, L. (2016). Forest density preferences of homebuyers in the wildland-urban interface. Forest Policy and Economics, 70, 56–66. https://doi.org/10.1016/j.forpol.2016.05.012.

25. Holmes, T.P., Murphy, E.A., Bell, K.P., Royle, D.D. (2011). Property value impacts of hemlock woolly Adelgid in residential forests. Forest Science 56(2):529-540. https://www.srs.fs.usda.gov/pubs/ja/2010/ja_2010_holmes_004.pdf. Accessed 2nd October 2022.

26. Hoover, F.-A., Price, J. I., & Hopton, M. E. (2020). Examining the effects of green infrastructure on residential sales prices in Omaha, Nebraska. Urban Forestry & Urban Greening, 126778. https://doi.org/10.1016/j.ufug.2020.126778.

27. Jensen, C. U., Panduro, T. E., Lundhede, T. H., von Graevenitz, K., & Thorsen, B. J. (2021). Who demands peri-urban nature? A second stage hedonic house price estimation of household’s preference for peri-urban nature. Landscape and Urban Planning, 207, 104016. https://doi.org/10.1016/j.landurbplan.2020.104016.

28. Jiao, M., Xue, H., Yan, J., Zheng, Z., Wang, J., Zhao, C., Zhang, L., & Zhou, W. (2021). Tree abundance, diversity and their driving and indicative factors in Beijing’s residential areas. Ecological Indicators, 125, 107462. https://doi.org/10.1016/j.ecolind.2021.107462.

29. Jiao, L., & Liu, Y. (2010). Geographic Field Model based hedonic valuation of urban open spaces in Wuhan, China. Landscape and Urban Planning, 98(1), 47-55. https://doi.org/10.1016/j.landurbplan.2010.07.009.

30. Jim, C. Y., & Chen, W. Y. (2010). External effects of neighbourhood parks and landscape elements on high-rise residential value. Land Use Policy, 27(2), 662-670. https://doi.org/10.1016/j.landusepol.2009.08.027.

31. Jo-Black, K., & Richards, M. (2020). Eco-gentrification and who benefits from urban green amenities: NYC’s high Line. Landscape and Urban Planning, 204, 103900. https://doi.org/10.1016/j.landurbplan.2020.103900.

32. Jun, M.-J., & Kim, H.-J. (2017). Measuring the effect of greenbelt proximity on apartment rents in Seoul. Cities, 62, 10-22. https://doi.org/10.1016/j.cities.2016.11.002.

33. Kadish, J., & Netusil, N. R. (2012). Valuing vegetation in an urban watershed. Landscape and Urban Planning, 104(1), 59–65. https://doi.org/10.1016/j.landurbplan.2011.09.004.

34. Kim, J.-H., Li, W., Newman, G., Park, S. Y., & Kil, S.-H. (2016). The influence of urban landscape spatial patterns on single-family housing prices. Environment and Planning B: Urban Analytics and City Science, 45(1), 26–43. https://doi.org/10.1177/0265813516663932.

35. Larson, E., & Perrings, C. (2013). The value of water-related amenities in an arid city: The case of the Phoenix metropolitan area. Landscape and Urban Planning, 109(1), 45–55. https://doi.org/10.1016/j.landurbplan.2012.10.008.

36. Łaszkiewicz, E., Heyman, A., Chen, X., Cimburova, Z., Nowell, M., & Barton, D. N. (2022). Valuing access to urban greenspace using non-linear distance decay in hedonic property pricing. Ecosystem Services, 53, 101394. https://doi.org/10.1016/j.ecoser.2021.101394.

37. Łaszkiewicz, E., Czembrowski, P., & Kronenberg, J. (2019). Can proximity to urban green spaces be considered a luxury? Classifying a non-tradable good with the use of hedonic pricing method. Ecological Economics, 161, 237–247. https://doi.org/10.1016/j.ecolecon.2019.03.025.

38. Li, H., & Wei, Y. D. (2020). Spatial inequality of housing value changes since the financial crisis. Applied Geography, 115, 102141. https://doi.org/10.1016/j.apgeog.2019.102141.

39. Li, X., Holmes, T. P., Boyle, K. J., Crocker, E. V., Nelson, C. D. (2019). Hedonic analysis of forest pest invasion: the Case of Emerald Ash Borer. Forests, 10(9), 820. https://doi.org/10.3390/f10090820.

40. Li, H., Wei, Y. D., Yu, Z., & Tian, G. (2016). Amenity, accessibility and housing values in metropolitan USA: A study of Salt Lake County, Utah. Cities, 59, 113–125. https://doi.org/10.1016/j.cities.2016.07.001.

41. Li, W., Saphores, J. D. M., & Gillespie, T. W. (2015). A comparison of the economic benefits of urban green spaces estimated with NDVI and with high-resolution land cover data. Landscape and Urban Planning, 133, 105–117. https://doi.org/10.1016/j.landurbplan.2014.09.013.

42. Li, W., & Saphores, J. (2012). A spatial hedonic analysis of the value of urban land cover in the multifamily housing market in Los Angeles, CA. Urban Studies, 49(12), 2597–2615. https://doi.org/10.1177/0042098011429486.

43. Liang, X., Liu, Y., Qiu, T., Jing, Y., & Fang, F. (2018). The effects of locational factors on the housing prices of residential communities: The case of Ningbo, China. Habitat International. https://doi.org/10.1016/j.habitatint.2018.09.004.

44. Liebelt, V., Bartke, S., & Schwarz, N. (2019). Urban green spaces and housing prices: An alternative perspective. Sustainability, 11(13), 3707. http://dx.doi.org/10.3390/su11133707.

45. Liu, F., Min, M., Zhao, K., & Hu, W. (2020a). Spatial-temporal variation in the impacts of urban infrastructure on housing prices in Wuhan, China. Sustainability, 12(3), 1281. https://doi.org/10.3390/su12031281.

46. Liu, T., Hu, W., Song, Y., & Zhang, A. (2020b). Exploring spillover effects of ecological lands: A spatial multilevel hedonic price model of the housing market in Wuhan, China. Ecological Economics, 170, 106568. https://doi.org/10.1016/j.ecolecon.2019.106568.

47. Liu, G., Wang, X., Gu, J., Liu, Y., & Zhou, T. (2019). Temporal and spatial effects of a “Shan Shui” landscape on housing price: A case study of Chongqing, China. Habitat International, 102068. https://doi.org/10.1016/j.habitatint.2019.102068.

48. Luo, J., Zhai, S., Song, G., He, X., Song, H., Chen, J., Liu, H., Feng, Y. (2022) Assessing inequity in green space exposure toward a “15-minute city” in Zhengzhou, China: Using deep learning and urban big data. International Journal of Environmental Research and Public Health, 19(10), 5798. https://doi.org/10.3390/ijerph19105798.

49. Ma, S., Kumakoshi, Y., Koizumi, H., Yoshimura, Y. (2022). Determining the association of the built environment and socioeconomic attributes with urban shrinking in Yokohama City. Cities, 120, 103474. https://doi.org/10.1016/j.cities.2021.103474.

50. Ma, Y., & Gopal, S. (2018). Geographically weighted regression models in estimating median home prices in towns of Massachusetts based on an urban sustainability framework. Sustainability, 10(4). https://doi.org/10.3390/su10041026.

51. McPherson, E. G., Xiao, Q., van Doorn, N. S., de Goede, J., Bjorkman, J., Hollander, A., Boynton, R. M., Quinn, J. F., & Thorne, J. H. (2017). The structure, function and value of urban forests in California communities. Urban Forestry & Urban Greening, 28, 43–53. https://doi.org/10.1016/j.ufug.2017.09.013.

52. Mei, Y., Zhao, X., Gao, L., & Lin, L. (2018a). Capitalization of urban green vegetation in a housing market with poor environmental quality: Evidence from Beijing. Journal of Urban Planning and Development, 144(3). https://doi.org/10.1061/(ASCE)UP.1943-5444.0000458.

53. Mei, Y., Hite, D., & Sohngen, B. (2018b). Estimation of house price differential of urban tree cover: An application of sample selection approach. Applied Economics, 50(25), 2804–2811. https://doi.org/10.1080/00036846.2017.1409419.

54. Mei, Y., Hite, D., & Sohngen, B. (2017). Demand for urban tree cover: A two-stage hedonic price analysis in California. Forest Policy and Economics, 83, 29-35. https://doi.org/10.1016/j.forpol.2017.05.009.

55. Melichar, J., & Kaprová, K. (2013). Revealing preferences of Prague’s homebuyers toward greenery amenities: The empirical evidence of distance-size effect. Landscape and Urban Planning, 109(1), 56–66. doi:10.1016/j.landurbplan.2012.09.

56. Netusil, N. R., Chattopadhyay, S., & Kovacs, K. F. (2010). Estimating the demand for tree canopy: A second-stage hedonic price analysis in Portland, Oregon. Land Economics, 86(2), 281-293. https://doi.org/10.3368/le.86.2.281.

57. Noor, N. M., Asmawi, M. Z., & Abdullah, A. (2015). Sustainable urban regeneration: GIS and hedonic pricing method in determining the value of green space in housing area. Procedia - Social and Behavioral Sciences, 170, 669–679. https://doi.org/10.1016/j.sbspro.2015.01.069.

58. Pandit, R., Polyakov, M., & Sadler, R. (2014). Valuing public and private urban tree canopy cover. Australian Journal of Agricultural and Resource Economics, 58, 453-470. https://doi.org/10.1111/1467-8489.12037.

59. Pandit, R., Polyakov, M., Tapsuwan, S., & Moran, T. (2013). The effect of street trees on property value in Perth, Western Australia. Landscape and Urban Planning, 110, 134-142. https://doi.org/10.1016/j.landurbplan.2012.11.001.

60. Panduro, T. E., & Veie, K. L. (2013). Classification and valuation of urban green spaces - A hedonic house price valuation. Landscape and Urban Planning, 120, 119–128. https://doi.org/10.1016/j.landurbplan.2013.08.

61. Park, J., Lee, D., Park, C., Kim, H., Jung, T., & Kim, S. (2017). Park accessibility impacts housing prices in seoul. Sustainability, 9(2), 185. https://doi.org/10.3390/su9020185.

62. Park, Y., & Kim, H. W. (2017). The cross-level impact of landscape patterns on housing premiums in micro-neighborhoods. Urban Forestry & Urban Greening, 24, 80-91. https://doi.org/10.1016/j.ufug.2017.03.020.

63. Plant, L., Rambaldi, A., & Sipe, N. (2017). Evaluating revealed preferences for street tree cover targets: a business case for collaborative investment in leafier streetscapes in Brisbane, Australia. Ecological Economics, 134, 238–249. https://doi.org/10.1016/j.ecolecon.2016.12.026.

64. Plant, L., Morrison, T., & Rambaldi, A. (2016). Street trees: Paying their way in property value benefits (G. Groening, G. Moore, J. Rayner, & E. Moore, Eds.; WOS:000391250200002; 1108, pp. 13–24). https://doi.org/10.17660/ActaHortic.2016.1108.2.

65. Piaggio, M. (2021). The value of public urban green spaces: Measuring the effects of proximity to and size of urban green spaces on housing market values in San José, Costa Rica. Land Use Policy, 109, 105656. doi:10.1016/j.landusepol.2021.105656.

66. Poudyal, N. C., Hodges, D. G., Fenderson, J., & Tarkington, W. (2010). Realizing the economic value of a forested landscape in a viewshed. Southern Journal of Applied Forestry, 34(2), 72-78. https://doi.org/10.1093/sjaf/34.2.72.

67. Price, J. I., McCollum, D. W., & Berrens, R. P. (2010). Insect infestation and residential property values: A hedonic analysis of the mountain pine beetle epidemic. Forest Policy and Economics, 12(6), 415–422. https://doi.org/10.1016/j.forpol.2010.05.004.

68. Qiu, W., Huang, X., Li, X., Li, W., & Zhang, Z. (2020). Investigating the impacts of street environment on pre-owned housing price in Shanghai using street-level images (J. Domenech & M. Vicente, Eds.; WOS:000759227700004; 29–39. https://doi.org/10.4995/CARMA2020.2020.11410.

69. Sander, H., Polasky, S., & Haight, R. G. (2010). The value of urban tree cover: A hedonic property price model in Ramsey and Dakota Counties, Minnesota, USA. Ecological Economics, 69(8), 1646–1656. https://doi.org/10.1016/j.ecolecon.2010.03.011.

70. Saphores, J. D., & Li, W. (2012). Estimating the value of urban green areas: A hedonic pricing analysis of the single family housing market in Los Angeles, CA. Landscape and Urban Planning, 104(3), 373–387. https://doi.org/10.1016/j.landurbplan.2011.11.012.

71. Seo, Y. (2020). Varying effects of urban tree canopies on residential property values across neighborhoods. Sustainability, 12(10). https://doi.org/10.3390/su12104331.

72. Tam, V. W. Y., Fung, I. W. H., Wang, J., & Ma, M. (2019). Effects of locations, structures and neighbourhoods to housing price: an empirical study in Shanghai, China. International Journal of Construction Management, 1–20. https://doi.org/10.1080/15623599.2019.1695097.

73. Tian, G., Wei, Y. D., & Li, H. (2017). Effects of accessibility and environmental health risk on housing prices: a case of Salt Lake County, Utah. Applied Geography, 89, 12–21. https://doi.org/10.1016/j.apgeog.2017.09.010.

74. Tran, T. J., Helmus, M. R., & Behm, J. E. (2020). Green infrastructure space and traits (GIST) model: Integrating green infrastructure spatial placement and plant traits to maximize multifunctionality. Urban Forestry & Urban Greening, 49, 126635. https://doi.org/10.1016/j.ufug.2020.126635.

75. Tuffery, L. (2017). The recreational services value of the nearby periurban forest versus the regional forest environment. Journal of Forest Economics, 28, 33-41. https://doi.org/10.1016/j.jfe.2017.04.004.

76. Votsis, A. (2017). Planning for green infrastructure: The spatial effects of parks, forests, and fields on Helsinki’s apartment prices. Ecological Economics, 132, 279–289. https://doi.org/10.1016/j.ecolecon.2016.09.029.

77. Wang, H., Hu, Y., Tang, L., & Zhuo, Q. (2020). Distribution of urban blue and green space in Beijing and its influence factors. Sustainability, 12(6). https://doi.org/10.3390/su12062252.

78. Wang, H.-F., Cheng, X.-L., Nizamani, M.M., Balfour, K., Da, L., Zhu, Z.-X., & Qureshi, S. (2020). An integrated approach to study spatial patterns and drivers of land cover within urban functional units: A multi-city comparative study in China. Remote Sensing, 12(14), 2201. https://doi.org/10.3390/rs12142201.

79. Wang, H.-F., Qiu, J.-X., Breuste, J., Ross Friedman, C., Zhou, W.-Q., & Wang, X.-K. (2013). Variations of urban greenness across urban structural units in Beijing, China. Urban Forestry & Urban Greening, 12(4), 554–561. https://doi.org/10.1016/j.ufug.2013.05.004.

80. Wang, H.-F., Qureshi, S., Qureshi, B. A., Qiu, J.-X., Friedman, C. R., Breuste, J., & Wang, X.-K. (2016). A multivariate analysis integrating ecological, socioeconomic and physical characteristics to investigate urban forest cover and plant diversity in Beijing, China. Ecological Indicators, 60, 921–929. https://doi.org/10.1016/j.ecolind.2015.08.015.

81. Wen, H., Zhang, Y., & Zhang, L. (2015). Assessing amenity effects of urban landscapes on housing price in Hangzhou, China. Urban Forestry & Urban Greening, 14(4), 1017–1026. https://doi.org/10.1016/j.ufug.2015.09.013.

82. Wu, C., Ye, X., Du, Q., & Luo, P. (2017). Spatial effects of accessibility to parks on housing prices in Shenzhen, China. Habitat International, 63, 45–54. https://doi.org/10.1016/j.habitatint.2017.03.010.

83. Wu, J., Wang, M., Li, W., Peng, J., & Huang, L. (2015). Impact of urban green space on residential housing prices: Case Study in Shenzhen. Journal of Urban Planning and Development, 141(4), 05014023. https://doi.org/10.1061/(asce)up.1943-5444.0000241.

84. Xiao, Y., Li, Z., & Webster, C. (2016). Estimating the mediating effect of privately-supplied green space on the relationship between urban public green space and property value: Evidence from Shanghai, China. Land Use Policy, 54, 439-447. https://doi.org/10.1016/j.landusepol.2016.03.001.

85. Yu, Y., Zhang, W., Fu, P., Huang, W., Li, K., & Cao, K. (2020). The spatial optimization and evaluation of the economic, ecological, and social value of urban green space in Shenzhen. Sustainability, 12(5), 1844. https://doi.org/10.3390/su12051844.

86. Zambrano-Monserrate, M. A., Ruano, M. A., Yoong-Parraga, C., & Silva, C. A. (2021). Urban green spaces and housing prices in developing countries: A Two-stage quantile spatial regression analysis. Forest Policy and Economics, 125, 102420. https://doi.org/10.1016/j.forpol.2021.102420.

87. Zhang, Y., & Dong, R. (2018). Impacts of street-visible greenery on housing prices: evidence from a hedonic price model and a massive street view image dataset in Beijing. ISPRS International Journal of Geo-Information, 7(3). https://doi.org/10.3390/ijgi7030104.

88. Zhang, Y., Fu, X., Lv, C., & Li, S. (2021). The premium of public perceived greenery: A framework using Multiscale GWR and deep learning. International journal of Environmental Research and Public Health, 18(13), 6809. https://doi.org/10.3390/ijerph18136809.

89. Zhu, Z.-X., Pei, H.-Q., Schamp, B. S., Qiu, J.-X., Cai, G.-Y., Cheng, X.-L., & Wang, H.-F. (2019). Land cover and plant diversity in tropical coastal urban Haikou, China. Urban Forestry & Urban Greening, 44, 126395. https://doi.org/10.1016/j.ufug.2019.126395.
